# Immunoglobulin A, an Active Liaison for Host-Microbiota Homeostasis

**DOI:** 10.3390/microorganisms9102117

**Published:** 2021-10-08

**Authors:** Ahmed A. Abokor, Grant H. McDaniel, Rachel M. Golonka, Connor Campbell, Sreya Brahmandam, Beng San Yeoh, Bina Joe, Matam Vijay-Kumar, Piu Saha

**Affiliations:** 1 Department of Physiology & Pharmacology, University of Toledo College of Medicine and Life Sciences, Toledo, OH 43614, USA; ahmed.abokor@rockets.utoledo.edu (A.A.A.); Rachel.Golonka@rockets.utoledo.edu (R.M.G.); bengsan.yeoh@utoledo.edu (B.S.Y.); Bina.Joe@utoledo.edu (B.J.); MatamVijay.Kumar@UToledo.edu (M.V.-K.); 2 College of Medicine, University of Toledo, Toledo, OH 43614, USA; Grant.Mcdaniel@rockets.utoledo.edu (G.H.M.); Connor.Campbell@rockets.utoledo.edu (C.C.); Sreya.Brahmandam@rockets.utoledo.edu (S.B.)

**Keywords:** secretory IgA, gut homeostasis, IgA deficiency, polymeric immunoglobulin receptor (pIgR), mucosal immunology, B cells

## Abstract

Mucosal surfaces in the gastrointestinal tract are continually exposed to native, commensal antigens and susceptible to foreign, infectious antigens. Immunoglobulin A (IgA) provides dual humoral responses that create a symbiotic environment for the resident gut microbiota and prevent the invasion of enteric pathogens. This review features recent immunological and microbial studies that elucidate the underlying IgA and microbiota-dependent mechanisms for mutualism at physiological conditions. IgA derailment and concurrent microbiota instability in pathological diseases are also discussed in detail. Highlights of this review underscore that the source of IgA and its structural form can dictate microbiota reactivity to sustain a diverse niche where both host and bacteria benefit. Other important studies emphasize IgA insufficiency can result in the bloom of opportunistic pathogens that encroach the intestinal epithelia and disseminate into circulation. The continual growth of knowledge in these subjects can lead to the development of therapeutics targeting IgA and/or the microbiota to treat life threatening diseases.

## 1. Introduction

Immunoglobulins (Ig), also known as antibodies, are large Y-shaped glycoproteins produced by plasma cells. Ig are involved in the clearance and neutralization of foreign particles in the body by identifying, binding, and eliminating specific bacterial, fungal and viral antigens. Out of the five Ig in the body, immunoglobulin A (IgA) is the second most abundant antibody found in circulation and the predominant antibody generated in mucosal secretions, whose primary function is to defend mucosal surfaces (e.g., gastrointestinal and respiratory tracts) from pathogen invasion [1,2,3]. In this review, we begin by explaining in-depth the production of IgA through both T cell dependent and independent pathways, the divergent IgA subclasses, and the differential functions between circulating and secretory IgA (SIgA). We then expand on how the robust reactivity between SIgA and the host microbiome can dictate species fitness and overall gut homeostasis. We follow by delineating how a disruption in the IgA-microbiome axis contributes to pathophysiological conditions such as colitis, colorectal cancer, and nephropathy. In addition, altered IgA responses to the gut microbiota have even been documented in asthma, food allergies and obesity, further illustrating the need for additional studies regarding IgA outside the scope of immunological diseases [4,5]. As IgA is the main antibody in maternal milk, we also discuss how this first source of antibody-mediated immunity protects infants from necrotizing enterocolitis. Overall, continual research to decipher the IgA-dependent mechanisms for microbiota-immune stability has the potential to be therapeutically exploited in the defense against pathological diseases. 

## 2. Immunoglobulins: The Basics

Ig belong to various classes and subclasses (isotypes) that differ in their structure, target specificity and localization. In the cognominal immunoglobulin superfamily (IgSF), Ig are structurally constructed with two pairs of heavy (H) and light (L) chains that constitute the crystallizable fragment (F_c_) and the antibody-binding fragment (F_ab_), respectively [6]. In the Y-shaped antibody, the F_c_ region frames the tail/trunk whereas the F_ab_ region composes the arms/branches. Each fragment contains an NH2-terminal “variable” (V) domain consisting of three hypervariable loops termed complementarity determining regions (CDRs) [7]. As CDRs are in direct contact with antigens, they often undergo extensive and frequent hypermutations, which enable Ig to recognize a near limitless number of different antigens for their adaptive immune function [8]. Both fragments also contain one (F_ab_ fragment) or more (F_c_ fragment) COOH-terminal “constant” (C) domains [7]. When the F_c_ fragment has three C domains, a middle *hinge* region, rich in proline and cysteine, is required to space out the first and second C domains [7]. Importantly, delicate distinctions in C domains of the F_c_ fragment are what differentiates the five Ig classes: IgM, IgG, IgD, IgE, and IgA. Naïve B cells first produce IgM and IgD with other isotypes produced later upon maturation of B cells via class switch recombination. In this process, only the C domain in the F_c_ region is juxtaposed, so that the V domain in the F_ab_ fragment retains affinity toward recurring antigens. In the next section, we discuss how the basic IgM and IgD classes switch to IgA in both T cell dependent and independent manners.

## 3. IgA: A Unique Structural and Functional Antibody

### 3.1. IgA Production in Germinal Centers

Gut-associated lymphoid tissue (GALT) comprises the secondary lymphoid organs named Peyer’s patches, mesenteric lymph nodes, and isolated lymphoid follicles found in the small intestine. These lymphoid organs are composed of three, interactive layers that partake in IgA production: the B cell-rich follicles, the T cell-rich interfollicular zones and a subepithelial dome rich in CD11c^+^ dendritic cells (DCs) that separate the epithelium from the follicles. There are both T cell-independent and dependent pathways for IgA production in the germinal centers. In the former, when a luminal antigen is present, specialized allograft inflammatory factor 1 (Aif1)-expressing microfold (M) cells lining the follicle-associated epithelium transport the antigen via transcytosis to the DC-rich subepithelial dome [9,10,11]. DCs expressing high levels of lysozyme then capture the luminal antigen for its activation [12,13]. Activated B cells that have travelled from the follicle region to the subepithelial dome via the chemokine receptor CCR6 can then interact with these DCs [14]. During this interaction, DCs undergo integrin αvβ8-mediated activation and secrete transforming growth factor-β (TGFβ) [14,15,16]. This cytokine then promotes TGFβRII-SMAD signaling, followed by inducing the expression of activation-induced cytidine deaminase (AID, a DNA-editing enzyme) and gene promoters for the constant heavy chain ‘alpha’, collectively ensuing IgA class switching in B cells [14,16,17].

In contrast, IgA production can be induced in a T cell dependent manner. Antigen-carrying M cells can elicit Th1, Th17, and Th22 responses [18] and CCR6^+^ DCs can directly activate pathogen-specific T cells [19]. DCs can also migrate to the T cell-rich interfollicular zones and cue the priming of CD4^+^ helper T cells to become a subset of follicular helper T (T_FH_) cells [20]. There are currently two independent reports that provide separate mechanisms for this differentiation. In one approach, the loss of FOXP3 from CD4^+^ T cells allows the conversion to T_FH_ cells, and in the other, RORγ^+^ Th17 cells differentiate into T_FH_ cells in an IL-17 and IL-22-dependent, but IL-23-independent manner [21,22]. In favor of the latter, it was recently shown that commensal antigens activate macrophage-inducible C-type lectin receptor (Mincle) in lysozyme-expressing DCs, where the secretion of IL-6 and IL-23p19 initiated Th17 polarization through its regulation of IL-17 and IL-22 [23]. Following activation, CD4^+^ T_FH_ cells interact with B cells via the CD40 ligand (member of the tumor necrosis factor (TNF) family), which stimulates TGFβ and IgA producing plasma cells [21,22,24]. Importantly, T cell dependent IgA production in germinal centers subsequently undergoes somatic hypermutation and antigen affinity selection [25]. As these IgA^+^ B cells coexpress type 1 sphingosine-1-phosphate receptor (S1P_1_), this mediates their exit from the Peyer’s patches to enter the lamina propria for plasma cell maturation and polymeric IgA production [26,27].

There is a possibility that excess T_FH_ cells with altered phenotypes may cause dysregulated IgA production within the germinal centers. Kawamoto et al. observed this in programmed cell death-1 (PD1)-deficient mice, which produced IgA with reduced bacteria-binding capacity, emphasizing PD1 involvement in the differentiation of T_FH_ cells to be critical in the appropriate selection of IgA plasma cell repertoires [28]. In line with this, mice deficient for the ATP-gated ionotropic receptor P2X7 had T_FH_ cell expansion, accumulated *Lactobacillus*-specific secretory IgA, and metabolic dysfunction [29,30]. As such, it is noteworthy that TGFβ and group 3 innate lymphoid cells (ILC3) were independently found to restrain T_FH_ cell accumulation and thereby prevent B cell mediated autoimmunity [31,32]. While DCs have been well established in the induction of IgA-producing plasma cells, it is intriguing that gut specific macrophages and eosinophils are other myeloid cells capable of inducing IgA production through the promotion of tertiary lymphoid structures and Peyer’s patches, respectively [33,34].

### 3.2. IgA Production in Nongerminal Centers

Much evidence shows that unorganized, nongerminal centers in the lamina propria can also promote T cell independent, microbial-induced IgA class switching. The mechanism behind T cell independent IgA class switching in the lamina propria is rooted to resident DCs upregulating two ligand members of the TNF family, known as the B cell activating factor (BAFF, *alias* B lymphocyte stimulator (BLyS) or TNFSF 13b) and a proliferation-inducing ligand (APRIL, *alias* TNFSF 13a) [35]. BAFF and APRIL bind to two TNF receptors: B cell maturation antigen (BCMA) exclusively on CD5^+^ B1 cells and transmembrane activator, and calcium-modulator and cytophilin ligand interactor (TACI) found on both B and T cells [35,36]. In the presence of TGFβ alone, or with a microbial product like lipopolysaccharide (LPS), BAFF and APRIL activate class switching from the μ-heavy chain (IgM) antibody to the alpha constant heavy chain (IgA) antibody [35,37]. Accordingly, APRIL deficient mice had an impairment for T cell independent IgA class switch recombination but could produce IgA in response to T cell dependent antigens [38]. Notably, interleukin-10, another class switching recombination cytokine, can also stimulate IgG and IgA production through BAFF and APRIL [35]. Besides these class switching cytokines, chemokine CCL28 induces the migration of IgA Ab-secreting cells (ASCs) via CCR10. Mice deficient in CCL28 have significantly reduced ASCs in the lamina propria, an overgrowth of opportunistic pathogens like *Bacillus cereus* and *Enterococcus faecalis*, and more susceptibility to mucosal inflammation [39]. Aside from BAFF and APRIL-mediated class switching, ER-stressed mucosa via defective autophagy can induce T cell independent polyreactive IgA production through expansion of a subset of peritoneal B cells known as B1b cells [40]. 

Compared to the conventional T cell dependent antigens, CD103^+^ DCs capture molecular patterns from commensal and pathogenic bacteria inhabiting the gut microbiome and then trigger T cell independent IgA production [41]. The CD103^+^ DCs themselves can recognize and carry these antigens, in addition to goblet cells serving as a supplemental delivery system to pass on low molecular weight soluble antigens to CD103^+^ DCs in the lamina propria [42]. As most molecular patterns are recognized by Toll-like receptors, DCs undergo in situ inducible nitric oxide synthase (iNOS) signaling for T cell independent IgA production. This was apparent when Tezuka et al. observed iNOS-deficient mice to have significantly lower levels of serum IgA and IgG (with the other Ig classes unaffected) and impaired IgA production in the intestine [43]. Importantly, this study identified a specific, natural subset of iNOS/TNF-α producing DCs residing in the lamina propria to be responsible for BAFF/APRIL-dependent IgA synthesis, where mice deficient in other Toll-like receptors resulted in a defect for iNOS-dependent IgA production [43]. This study also demonstrated iNOS to be required for T cell-dependent IgA class switching as AID became defective and TGFβRII levels were significantly depleted in iNOS knockout mice [43], underscoring the absolute necessity for iNOS in whole body IgA production. It is important to mention that IgA^+^ cells can also express iNOS and TNF-α, essentially creating a positive feedback loop to support IgA levels in the gut [44]. 

To summarize, the Peyer’s patches and lamina propria in the small intestine are the central hubs for T cell dependent and independent homeostatic IgA production, respectively. It should be noted, however, that the colon has been reported to exhibit T cell independent IgA class switch recombination but only in the presence of organized lymphoid follicles [45]. Irrespective, DCs are the main antigen-presenting cells that can recognize both T cell dependent antigens and microbial molecular patterns to stimulate intestinal IgA production in either the Peyer’s patches or lamina propria, respectively. Besides the mentioned TNF ligands (CD40L, BAFF, and APRIL) and cytokines (TGFβ), other studies have showed additional environmental factors, like retinoic acid (RA) and other interleukins (e.g., IL-5, IL-6, IL-21), to be synergistically required for IgA synthesis [27,46]. Recently, it was discovered that the acute phase protein, serum amyloid A (SAA), acts as a transporter for retinol to intestinal myeloid cells via LDL receptor-related protein 1 (LRP1), and the conversion of retinol to RA promoted RA-dependent IgA synthesis. Indeed, the same study observed that mice lacking SAA or LRP1 were significantly deficient in IgA production and more susceptible to enteric infection, highlighting the necessary function of vitamin A metabolism in intestinal adaptive immunity [47]. Moreover, certain cytokines, such as IL-21, were found to augment IgA production in the presence of microbiotal antigens [46]. As T cell-independent IgA is made in response to the endogenous microbiota, IgA is designed to be relatively nonspecific and polyreactive. The topic of IgA reactivity toward bacteria will be discussed further in Section 4, as this interaction is critical in the gut homeostasis and pathogenesis of various diseases.

### 3.3. IgA Subclasses in Humans and Mice

The human genome encodes two IgA isotypes, IgA1 and IgA2 (and two IgA2 allotypes IgA2m1 and IgA2m2), whereas the mouse genome encodes a single IgA with multiple allotypes. The divergence between these isotypes and allotypes is due to differences in hinge length and composition, including distinct glycosylation patterns [48,49,50,51]. Below, we first discuss human IgA and then make comparisons with murine IgA. 

In terms of structural differences, human IgA1 displays an extended hinge region consisting of two eight-amino acid long duplicate sequences (one per light chain) that is absent in IgA2. This amino acid sequence is the recognition site for IgA1-specific proteases and, depending on the bacterial IgA1 protease enzyme type, it may cleave one specific peptide bond within one of the duplicate sequences, but not at the equivalent site of the other duplicate sequence [52]. The lack of these 16 amino acids in human IgA2 makes this antibody inadvertently resistant to proteolysis. Another major difference between human IgA1 and IgA2 is its preference to be either monomeric or dimeric, respectively, and this can vary based on localization within the body. In circulation, human IgA is predominately monomeric with a ratio of 9:1 IgA1 to IgA2 [50]. At mucosal sites, human IgA is prevalent in a secretory, dimeric form and the proportions between dimeric IgA1 and IgA2 varies per site: 80 to 90% IgA1 in nasal and male genital secretions, 60% IgA1 in saliva, and 60% IgA2 in colonic and female genital secretions [50]. The prevalence for IgA2 class switching in the gut is mediated by intestinal epithelial cells that secrete APRIL after sensing commensal bacteria through Toll-like receptors [53]. As such, T cell independent IgA production in the local gut is essential to ensure appropriate mucosal immunity between host and microbiota [27,41].

Activated plasma cells localized in the mucosa produce dimeric human IgA via covalent linkage of the joining (J) chain to polypeptide extensions of the F_c_ regions on monomeric IgA in a process known as intracellular polymerization. Although dimeric IgA is predominantly produced through this process, J chain-containing trimeric and tetrameric IgA are also produced via intracellular polymerization when the cellular J chain is scarce [54]. The J chain serves as the ligand for the intestinal epithelial transmembrane protein known as polymeric immunoglobulin receptor (pIgR), which, upon receptor binding to the J chain, promotes endocytosis for the dimeric IgA (predominantly IgA2) across pIgR-expressing mucosal cells [55,56]. A portion of pIgR referred to as the transmembrane secretory component (SC) is cleaved from pIgR and becomes a component of the polymeric secretory IgA (SIgA) or, when detached, has antimicrobial properties [57]. Upon release into the lumen, SIgA binds to many receptors such as the IgA transmembrane receptor (FcαRI) expressed on myeloid cells such as eosinophils, neutrophils, macrophages, dendritic cells, and Kupffer cells (in depth discussion on SIgA function via FcαRI signaling can be found in Section 3.4) [58]. Of note is that SIgA utilizes ‘chaperones’ that assure antibody availability and stability. Recently, Xiong et al. demonstrated the marginal zone B and B-1 cell-specific protein (MZB1) to act as a chaperone by preventing the intracellular degradation of the IgA α-light-chain complexes, promoting J-chain binding to IgA, and increasing the secretion of dimeric IgA [59]. Intriguingly, in vitro studies indicated that the chaperone addition of SC limit SIgA proteolytic degradation [60] but was a silent bystander in terms of SIgA biological activity for antigen recognition and affinity [61]. *In vivo* studies further demonstrated that the SC did not improve antibody stability, but rather provided better tissue localization for the SIgA complex and localization of bacteria in damaged tissue [62], most likely due to its inherited bactericidal property.

When examining mouse IgA immunity, mice have only one IgA subclass and presumably use alternative receptors such as Fcα/μR, the transferrin receptor (CD71), and pIgR due to their lack of FcαRI (*alias* CD89) [63], which is the major IgA receptor in humans. Other significant differences include: (i) murine IgA is primarily monomeric whereas humans exhibit both mono- and dimeric forms; (ii) mice, but not humans, exhibit the B-1 B cell precursors of IgA plasma cells in the spleen; (iii) mice display three times less somatic hypermutations than humans; (iv) the germinal center boundary in mice is not well-defined when compared to humans, and (v) mice have low systemic IgA levels due to hepatic pIgR transporting serum IgA into bile, which then travels to the intestinal lumen [64]. It is noteworthy that human B cells have relatively low expression of Toll-like receptor 4 (TLR4, pattern recognition receptor for bacterial lipopolysaccharide) compared to mice [65], suggesting an increased demand for immune surveillance in these animals for regulating and maintaining their microbiota. This could signify why mice have a greater number of M cells and the additional pIgR expression on hepatocytes to deposit IgA in the intestinal lumen via the bile duct. In the next section, we further discuss how the function of systemic vs. SIgA mediates mucosal adaptive immunity.

### 3.4. Systemic vs. Mucosal IgA: A ‘Silent Panic Button’ vs. Robust Interaction

**Figure 1:**
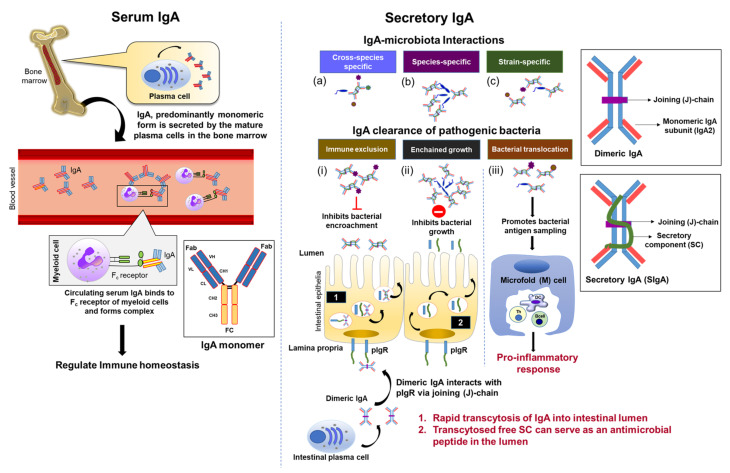
Structure and functions of serum and secretory IgA. In the left column, IgA, primarily monomeric IgA, is secreted by mature plasma cells in the bone marrow and enters systemic circulation. Circulating serum IgA forms immune complexes with transmembrane Fc receptors located on myeloid cells to induce downstream effector signaling necessary in maintaining immune homeostasis. In the right column, intestinal plasma cells produce dimeric IgA through divalent linkage of two IgA monomers to a joining (J)-chain. The J-chain binds to the secretory component (SC) of polymeric IgA receptors (pIgR) located on the basolateral surface of intestinal epithelial. IgA is rapidly transcytosed into the intestinal lumen as secretory IgA (SIgA). Free SC is also transcytosed into the lumen and serves as an antimicrobial peptide. Interacting with the gut microbiota, SIgA selectivity and reactivity can be categorized into either (**a**) cross-species (polyreactive) reactive against various bacterial species, (**b**) species-specific reactivity or (**c**) strain-specific reactivity. For pathogen removal, SIgA may (**i**) bind to and agglutinate bacteria, thus hindering microbial attachment and invasion of host intestinal epithelia, a process known as immune exclusion, (**ii**) prevent bacterial conjugation via enchained growth to limit bacterial proliferation, and (**iii**) expedite bacterial translocation through microfold (M) cells into Peyer’s patches for antigen sampling by resident dendritic cells (DC).

Naturally occurring systemic IgA is mainly immunoregulatory with little to no direct contact with microbes due, in part, to the sterile environment of blood. Previous studies have demonstrated the ability of serum IgA to effectively eliminate antigens without alerting the host immune system via inhibition of the complement system [66,67]. This allows serum IgA to act as a ‘silent panic button’ in the clearance of antigenic material from the body. However, it is worth noting that its structure is heavily involved in the general anti-inflammatory nature of IgA1. When monomeric, nonantigen-carrying IgA1 interacts with the myeloid IgA F_c_ receptor, FcαRI, and then the Src homology region 2 domain-containing phosphatase-1 (SHP-1) is recruited in an ERK-dependent manner to the docking site named inhibitory immunoreceptor tyrosine-based activation motif (ITAM) [68,69,70]. When FcαRI and ITAM colocalize with the surrounding lipid rafts, their complex forms inhibisome clusters called ITAMi, and the resulting impaired downstream phosphorylation blocks immune responses [68,69,70,71]. Compared to the IgA-mediated immune tolerance in circulation, dimeric IgA2 from the lamina propria can translocate into the intestinal lumen as SIgA and anchor itself on the outer mucosal surface to robustly interact with the gut bacteria for proper immune-microbiota stability [72]. This collectively establishes both serum and mucosal IgA to have important involvement in immune function under homeostatic conditions (Figure 1).

In the scenario of local bacterial dissemination, when the front-line defense of SIgA is not sufficient, dimeric IgA2 opsonizes the antigens by cross-linking with the resident Fcα/μR^+^ follicular DCs and recruited Fcα/μR^+^ neutrophils [73,74]. When cross-linked, the Src kinase Lyn phosphorylates the tyrosine within the associated ITAM and this promotes the recruitment of kinases/growth factors that stimulate the immune cells that are associated with phagocytosis, respiratory burst, and secretion of inflammatory cytokines [68,69,75,76,77]. Simultaneously, leukotriene B4 (LTB4) is secreted to act as a chemotaxis signal for more neutrophil recruitment to the site of infection, thus creating a positive feedback loop to eliminate invading pathogens [78,79]. In cases when bacterial infection and dissemination are severe enough to reach portal vein circulation, serum IgA opsonizes the antigen, cross-links with Kupffer cells (resident liver macrophages), and induces a pro-inflammatory response [80]. It is important to note that the natural per se anti and proinflammatory effector function of dimeric IgA2 and monomeric IgA1, respectively, were recently shown to be attributed to their different glycosylation profiles. Both antibodies contain several *N*-glycosylation sites, but only IgA1 has multiple *O*-glycosylation sites and therefore, has more terminal sialic acid per glycan. Steffen et al. reported that desialylation via neuraminidase treatment increased the proinflammatory capacities of IgA1 that mirrored IgA2 [49]. As circulating glycosylated IgA can contribute to the progress of various autoimmune diseases, targeting autoantibody glycosylation could be a potential therapeutic strategy. 

In summary, if we view the body as a fortress protecting against pathogen invaders, SIgA act as a ‘blockading wall’ in collaboration with the intestinal epithelia as an indispensable first line of defense to neutralize microbes. When bacteria invade past SIgA and breach the mucosal layer, dimeric IgA serves as the second line of innate mucosal immune defense, and then the teamwork of serum IgA and Kupffer cells becomes the third and last line of defense to eliminate pathogens, should they enter circulation. As SIgA is the most abundant antibody in the human body, the remainder of the review focuses on how SIgA provides immune protection through its interaction with the microbiota, and how malfunction in this system can lead to debilitating diseases.

## 4. SIgA: A Dynamic and Versatile Ally in Host-Microbiota Interactions

The human gastrointestinal tract is comprised of an estimated 100 trillion microorganisms, which outnumber our somatic and germ cells 10 to 1 in population quantity, effectively making us more ‘microbe than man’ [81]. The most densely microbe-populated portion of the intestine is the large intestine, and it is primarily colonized by two distinct phyla: *Firmicutes* and *Bacteroidetes*. A ratio between these two divisions, called the F/B ratio, serves as a putative marker for gut homeostasis in an individual [82,83]. Throughout our lives, the microbiota shapes both our innate and adaptive immune systems, where the greatest variability in bacterial colonization during the first three years of life are the most crucial time points [84]. We have already discussed that T cell independent SIgA synthesis is advanced by means of microbial stimulation, which could be thought of as a purposeful act to create a mutualistic environment between host and microbiota [85]. In the following sections, we describe the mechanisms for the generation of different SIgA reactive types and how these subgroups of SIgA recognize their bacterial targets for clearance. 

### 4.1. SIgA Is Selectively Reactive against the Gut Microbiota

SIgA interacts with the microbiota in maintaining homeostasis, with homeostatic properties largely dependent on the specificity of the antibody against various microbiota consortia. In the human gut, it is estimated that a single bacterium is coated with nearly 19,000 SIgA molecules, and this number increases to roughly 60,000 molecules for SIgA-coated bacterium in mice [86]. These antibody-microbiota interactions can be distinctly grouped into three categories based on SIgA reactivity: (i) cross-species, (ii) species-specific, and (iii) strain-specific reactivity [2] (Figure 1). Cross-species reactive SIgA refer to IgA antibodies with the capability of binding various unrelated bacterial taxa, and are typically polyreactive in that they are able to bind structurally distinct antigens (e.g., LPS, CpG). However, it was recently identified that SIgA somatic hypermutations, rather than polyreactivity, confer cross-species binding and high microbiota reactivity [87]. Cross-species reactive SIgAs innately arise within all naïve B cell subsets prior to plasma cell differentiation and bind to a broad subset of microbiota, which include most members in the phylum *Proteobacteria*, but these SIgAs largely lack binding to the predominant taxonomic groups *Bacteroidetes* and *Firmicutes* [88]. This is expected because, as it has been previously described, only 7% of intestinal SIgA present are cross-species reactive, while the majority of IgA are antigen-specific [89]. However, despite its low population, cross-species reactive SIgA plays a major role in the maintenance of microbiota diversity. In comparing BALB/c and C57BL/6 mouse strains, Fransen et al. found a genetic predisposition for certain mice to produce homeostatic polyreactive SIgAs, which were primarily involved in maintaining microbiota homeostasis [90]. Accordingly, this study demonstrated that all BALB/c mice displayed significantly more polyreactive SIgAs when compared to C56BL/6 mice irrespective of vendor source or housing condition, and this correlated significantly with increased microbiota diversification in BALB/c mice [90]. Notwithstanding, the same group also observed that germ-free (GF) BALB/c mice, but not C57BL/6 mice, possessed considerably elevated quantities of microbial-independent, innate SIgAs at baseline, which were further augmented following microbiota colonization, indicating a genetic predisposition involved in the innate proclivity for BALB/c mice to produce these antibodies [90]. 

Species-specific reactive SIgAs refer to IgA antibodies that bind exclusively to different bacterial species present in the gut. While it is not exactly known how IgA discriminates against distinct bacterial species, it is widely believed that bacterial surface carbohydrate moieties play a significant role in IgA selectivity across taxonomic species [91]. In GF mice monocolonized with *Bacteroides thetaiotaomicron (B. theta),* a highly specific gut SIgA response was induced with low cross-reactivity to other closely related *Bacteroides* [92]. It was also determined that anti-*B. theta* IgA targeted proteins on the *B. theta* polysaccharide utilization locus (PUL), which indicated bacterial fructans as a potential epitope required for species specific SIgA selectivity [92]. Another study with a reversible *in vivo* germ-free colonization model further substantiated the high precision for species specific SIgA. In this model, GF mice were primed with a triple mutant of the auxotrophic *E. coli K-12* mutants (known as strain HA107), but as this strain cannot divide nor persist *in vivo*, the mice returned to their germ-free state within 72 h. Upon re-exposure to *E. coli* HA107, the GF mice produced a distinct mucosal SIgA response to this bacterial strain, whereas first time exposure to *Salmonella typhimurium* (*S. typhi*) did not elicit a species specific SIgA response in the pretreated *E. coli* HA107 GF mice [93]. In line with this, *E. coli* HA107-pretreated GF mice that were later colonized with a compositionally-defined microbiota consortia deficient in *E. coli* lacked SIgA with *E. coli*-binding capabilities, *albeit* total IgA production did not decrease [93]. This evidence suggested that species presence may be a prerequisite for SIgA species specificity. Interestingly, a functional analysis of B cell responses to the intestinal microbiota detected SIgA antibodies against *Prevotella copri* (a Gram-negative commensal bacterium linked to rheumatoid arthritis) in the plasma and feces of healthy individuals in a human cohort that apparently lacked *Prevotella* in their gut microbiota [94]. The researchers attributed this phenomenon to the individuals having prior exposure at some point to the bacteria, and potentially developing antibodies to *Prevotella* long after clearance from the system, which they characterized to be in line with other prior studies on the subject. 

Strain-specific SIgAs are IgAs that are selective to various genetic variants, or subtypes, within a bacterial species. It was recently demonstrated that mice monocolonized with *Bacteroides ovatus* elicited a robust mucosal SIgA response; however, it was determined that certain *B. ovatus* variants were more effective at inducing colonic IgA-secreting plasma cells than other variants, leading these particular subtypes to have the classification of IgA^high^—*B. ovatus* [95]. Similarly, upon examination of bacterial strains found in human feces, it was observed that specific strains of *Bifidobacteria* were able to induce large quantities of IgA in vitro, whereas standard strains were able only weakly to induce IgA [96]. Additionally, monocolonization of the *B. theta* type strain VPI-5482 elicited a strain-specific IgA repertoire attracted to the capsular polysaccharides found on *B. theta* VPI-5482, but not other *B. theta* strains [97]. Likewise, IgA prevented bacterial adherence and invasion of wild-type *S. typhi*, in contrast to mutant strains, on polarized epithelial cell monolayers *in vitro* because IgA recognized a specific carbohydrate epitope on the wild-type *S. typhi* [98]. These findings collectively highlight the multiplicity of specific epitopes able to be recognized by SIgA and signify IgA responses to employ a great polyreactive repertoire to bind an extensive, but taxonomically diverse, subset of microbiota.

### 4.2. SIgA-Mediated Gut Pathogen Clearance and Homeostatic Properties

SIgA, the predominant form of IgA, serves a dynamic role in both protecting the host from pathogens and shaping the gut microbiota composition to promote host-microbiota homeostasis [99] (Figure 1). Compared to monomeric IgA, polymeric SIgA poorly activates F_c_ receptors for adequate downstream effector signaling [100]. For this reason, SIgA has developed several mechanisms that exploit its cross-linking ability and the intestinal environment for potent elimination of pathogens. The first mechanism for SIgA-mediated microbe neutralization is a process known as immune exclusion, which aims to intercept microbial access to the intestinal epithelium in a stepwise fashion: (i) antibody agglutination and cross-linkage; (ii) pathogen entrapment in mucus, and (iii) removal through peristalsis [101]. In this regard, SIgA acts more as a “blockading wall” to inhibit the translocation of microbes from the lumen to the blood. For instance, SIgA could prevent systemic infection in mice orally inoculated with *S. typhimurium*, but SIgA was unable to prevent bacteremia and systemic infection after intraperitoneal challenge of the same bacterium [102]. Later findings demonstrated that immune exclusion by SIgA was specifically designated within the mucosa. Another in vivo study substantiated immune exclusion of *Shigella* to require glycosylated residues on the secretory component of IgA for suitable localization of antibody molecules and optimal prevention of infection in the mucosa [62]. Moreover, IgA-mediated immune exclusion is not exclusive to bacterial pathogens but occurs also for commensal fungi. Recently it was discovered that SIgA can also target cell-surface adhesion proteins responsible for hyphae adherence and invasion of host cells by *Candida albicans* to prevent attachment and subsequent infection in humans [103]. 

A limitation to immune exclusion is that it is only efficient at high pathogen densities, unlike in typical infections. For this reason, another mechanism of SIgA-mediated pathogen elimination known as “enchained growth” has recently been proposed to be relatively effective at lower pathogen quantities. SIgA-mediated enchained growth works by enchaining and segregating bacterial plasmid donor and recipient clones to prevent conjugative plasmid transfer [104]. Enchained growth is finite, however, and clustered pathogen growth, *albeit* restricted, can occur until a certain size is obtained and then broken to form subclusters comprising of closely related bacteria [105]. A drawback to enchained growth is that it is most effective against fast-growing bacteria, as observed in a computational study by Bansept et al. on SIgA-mediated enchained growth in *Salmonella* spp. [106]. Indeed, the group noted that bacteria with high growth rates replicated prior to the linkage between daughter bacteria breaking, and generated larger cluster sizes, whereas bacteria with slower growth and replication rates had high probability in suffering cluster breaks earlier and escape SIgA enchained growth upon replication [106]. 

In addition to the above two mechanisms, SIgAs have an exclusive function called ‘coating’ to increase bacterial translocation in Peyer’s patches, which inadvertently improves resident DCs in their antigen sampling and activation [107,108]. For example, SIgA-coated *Shigella flexneri* was found to be rapidly transcytosed into Peyer’s patches and internalized by DCs, whereas uncoated *S. flexneri* was unable to penetrate the intestinal epithelium [107]. SIgA coating was reported to be dominant toward commensal bacteria in the small intestine, which, in turn, assisted resident colonization, whereas IgA-free bacteria were mostly indigenous to the colon [109]. Importantly, this ‘coating’ action of SIgA seems to be highly regulated as <5% of SIgA are utilized for bacterial coating despite sufficient SIgA available to coat nearly the entire microbiota population [86]. This can be inferred as an essential mechanism to sustain mutualism with the gut microbiota, whereas in disease conditions SIgA coating becomes more prevalent (see Section 5). Impressively, IgG and IgM have little capacity to coat anaerobic bacteria, emphasizing SIgA to be the main responsive immunological component against gut antigens [110]. 

SIgA has been found to be largely influential in maintaining gut homeostasis by reshaping the gut microbiota composition to promote gut symbiont growth and suppress pathogenic bacteria proliferation. For instance, SIgA that specifically coats mucus-resident symbiont *B. theta* has been shown to upregulate a cluster of genes provisionally named, Mucus-Associated Functional Factors (MAFFs), which function to ensue symbiosis amongst the *Firmicutes* and can also provide protection against chemically-induced colitis in mice [111]. Moreover, SIgA is critical for robust mucosal colonization and single-strain stability of commensal *Bacteroides fragilis* via commensal colonization factor (*ccf*)-mediated upregulation of capsular polysaccharides to attract IgA binding [112]. Intriguingly, *B. fragilis* possess endoglycosidase activity, and can therefore exploit the complex N-glycans heavily decorated on SIgA for necessary symbiotic bacteria growth [113]. Moreover, mucosal IgA maintains microbiota homeostasis by limiting commensal fungi proliferation, as SIgA has been observed to be reactive against *Candida glabrata*, *Candida albicans*, *Saccharomyces cerevisiae*, and *Candida tropicalis* found in human feces [103]. 

Aside from promoting beneficial commensal bacteria, SIgA facilitates healthy biodiversity in the gut microbiota starting at birth. A recent study by Fehr et al. found breast milk to transfer certain bacteria including *Streptococcus* spp. and *Veillonella dispar*, which contributed to the overall variation in offspring microbiota [114]. Another recent article by Donaldson et al. indicated that certain human commensals, such as *B. fragilis*, purposely alter the epithelial architecture to attract IgA-coating, which ultimately helps in the bacterium’s colonization within a specific mucosal niche [112]. Computational modeling by McLoughlin et al. further supports the concept that IgA is essential for microbial adhesion to the epithelial surface and can concurrently remove those bacteria for clearance to maintain appropriate diversity [115]. Comparatively, mice deficient in recombination activating protein-1 (RAG-1), which lack adaptive immunity, displayed considerably reduced diversity in the bacterial community when compared to their wild-type and heterozygous littermates [116]. In line with this, mice deficient in either only B or only T cells had altered bacterial communities that lacked diversity [116]. It was shown that the differentiation of Foxp3^+^ T cells into T_FH_ and follicle regulatory (T_FR_) cells in the germinal centers regulated both the quality and quantity of SIgA. Furthermore, the antibodies had various binding affinities that coated a moderate portion of the gut microbiota with the intention to maintain, not eliminate, the microbes for diversity [116]. This interaction between host SIgA and bacteria facilitated additional host immune responses in the gut to produce a symbiotic regulatory loop to maintain gut homeostasis [116]. Interestingly, *Clostridia* belonging to clusters IV and XIVa in the phylum *Firmicutes* were observed to be potent inducers of Foxp3^+^ T cells necessary for diversified IgA production [117,118]. Reciprocally, T cell dependent IgA responses to the symbiont *Akkermansia muciniphilia* provides “bystander protection” against enteric infections to further promote gut health and maintain homeostasis [119]. 

Even though the gut homeostatic functions of SIgA remain subtle, the extent of IgA responses, and the requirement to secrete mucosal IgA antibodies, supports its functional significance. Though it is not clear whether IgA antibodies may have either advantageous or deleterious effects on IgA-targeted microbes, the constitutive presence of IgA-coated commensals endorses that any harmful effects are not generally sufficient to cause elimination. In fact, IgA binding to bacterial capsular polysaccharides may be suppressed by some microbiota species to allow mucus layer attachment, thereby preventing a niche invasion from competing species [112,120].

## 5. Defects in IgA-Microbiota Axis Lead to Pathological Diseases

As emphasized in the earlier sections of this review, IgA plays a significant role in the immune system due to its structural significance, secretion, glycosylation, localization and receptor interactions. We also highlighted the ways in which IgA strongly influences the microbiota composition and its related gene expressions of various commensal microorganisms. Importantly, continuing research underscores the microbiota composition to be an implicated etiological factor in an increasing number of diseases, including gastroenterological (e.g., colitis), allergies, asthma, and metabolic disease [121]. As dysfunctions in IgA biology can also lead to multiple types of pathologies, it can be reasoned that a defect in the IgA-microbiota axis could explain the development of various diseases (Table 1). Herein, we discuss the literature that supports impaired IgA can lead to various pathologies regulated by microbiota.

### 5.1. IgA Deficiency in Autoimmunity

Undetectable levels of serum IgA involve quantities that are below 7 mg/dL. This clinical manifestation of human IgA deficiency is termed selective IgA deficiency (SIgAD) [122]. SIgAD is an interesting pathology because it is the most common primary immunodeficiency where the remaining Ig levels are normal. As can be expected, 20–30% of SIgAD patients suffer from autoimmune diseases [122,123]. Alarmingly, a cohort study by Jorgensen et al. showed that first-degree relatives of SIgAD patients had a 10% occurrence of autoimmune diseases, which is twice the estimated 5% in the general population [123]. Recalling how circulating IgA initiates anti-inflammatory signals through FcαRI-ITAMi (Section 3.4), the absence of IgA would then allow inflammatory responses to persist with no restraint, causing autoimmune disease development [124]. It is important to note that even when either B cells or IgA is absent, the intestinal epithelia can launch other protective defenses e.g., inducing the interferon-inducible immune response pathway, but only when the microbiota are present [125].

There are conflicting reports concerning whether human SIgAD is associated with substantial changes in gut microbial ecology. In a study from Fadlallah et al., their metagenomics analysis suggested minor perturbation in the microbiota, where IgA deficiency caused the expected pathobiont expansion but caused a less than expected depletion in some classic beneficial symbionts [126]. One explanation they had for this phenomenon was that the partial compensatory response in IgM levels could preserve microbiota diversity [127]. Contrary to this hypothesis, a later report by Catanzaro et al. showed that SIgAD patients still exhibit significant gut microbiota dysbiosis even with the compensatory IgM response [128]. This study uncovered that IgM had less specificity toward the commensals, and consequently coated a larger subset of the microbial species [128]. Interestingly, a recent study showed a compensatory IgG response in systemic circulation for SIgAD patients, where the IgG had antimicrobial properties toward commensals [129]. Another metagenomic study demonstrated that SIgAD patients had reduced microbial diversity but were enriched with opportunistic bacteria like *Escherichia coli* [130]. As with humans, the mouse model of IgA deficiency (IgA^−/−^) resulted in spontaneous inflammation specifically in the ileum, and segmented filamentous bacteria (SFB) were noted to bloom in IgA^−/−^ mice within the inherited dysbiotic microbiota [131]. As studies linking SIgAD and the gut microbiota are only a few years old, more research is needed to uncover what effects these have on the autoimmune disease progression. 

Besides SIgAD, there is a condition named common variable immunodeficiency (CVID) from ineffective antibody production, primarily IgG and IgA, due to a generalized B cell defect [132,133]. In addition, there is Omenn syndrome (OS) caused by hypomorphic RAG mutations, and this inadvertently results in IgA deficiency [134]. CVID and OS patients automatically have a greater risk in contracting bacterial infections, but they can also have noninfectious autoimmune complications e.g., inflammatory bowel disease and enteropathy. Despite minimal studies on the subject, it is hinted that unstable gut microbes do play a part in the autoimmune reactions seen in CVID and OS patients [133,134,135,136]. The following sections expand in detail on the link between IgA and microbiota in inflammatory and infectious diseases. 

### 5.2. IgA-Microbiota in Necrotizing Enterocolitis 

Necrotizing enterocolitis (NEC) is the most serious and common intestinal diseases in vulnerable infants. The risk for NEC in premature newborns increases in those with moderate to very low body weight, and the mortality rate is estimated to be 20–30% for the latter infants [137,138]. Contributing factors to NEC progression are focused on intestinal immaturity and improper microbial colonization in the neonatal period. NEC does not typically appear until 8-10 days after birth, which is when the gut is being colonized by facultative anaerobes from *Proteobacteria* and *Firmicutes* phyla [139]. Alarmingly, the immediate use of antibiotics when premature infants enter the neonatal intensive care unit (NICU) could perturb appropriate bacterial colonization and, therefore, cause NEC [140,141]. In essence, bacteria invade the intestinal wall to cause localized infection, and this is followed by epithelial injury, Paneth cell depletion, compromised barrier function, inflammation, necrosis, bacteremia and endotoxemia [137,138]. To study NEC in rodents, postnatal disruption in Paneth cell development via dithizone or diphtheria toxin are two of the most widely used approaches [142,143,144]. 

Germ-free studies confirm that the gut microbiota is a required element in NEC [145]. Importantly, microbial diversity appeared almost nonexistent in stool samples of NEC patients; this was signified with no more than seven poor colonizing species, but the families *Enterobacteriaceae* and γ-*Proteobacteria* were highly abundant [140,143,146]. Metagenomic and sequencing studies identified *Klebsiella* spp. such as the cytotoxin-producing *Klebsiella oxytoca* (notably in the *Enterobacteriaceae* family) to be substantial early colonizers in premature infants before NEC onset [147,148]. A recent study by Shaw et al. found a dichotomous microbiota profile in NEC infants where cases had high levels of LPS-expressing organisms (recognized by TLR4) but low levels of CpG DNA (recognized by TLR9) in the bacterial genomes [149]. These authors presented the hypothesis of two aberrant Toll-like receptor signals: (i) TLR4 overstimulation and (ii) TLR9 silence, which would normally counteract TLR4 [149]. Besides the already mentioned bacteria, there are conflicting reports as to whether *Clostridia* spp. are prevalent or depleted in NEC cases [140,150], but a report by La Rosa et al. demonstrated the highly regulated maturation progression from *Bacilli* to γ-*Proteobacteria* to *Clostridia* in the microbiota of premature infants [151]. Importantly, the same study showed that this nonrandom, sequential order of bacterial colonization is consistent, but can have different paces depending on factors like antibiotics, vaginal vs. caesarian birth, age, and breast milk vs. formula feeding [151]. 

In a Paneth cell disruption NEC rodent model, the addition of formula feeding exacerbated intestinal injury independent of gut microbial dysbiosis [152]. This result emphasized the caution needed for preterm infant feeding. Comparatively, maternal milk was found to substantially lower NEC incidence [153], suggesting an antimicrobial component in mother’s milk responsible for the protection. Of relevance to this review, dimeric and polymeric, but only minor traces of monomeric IgA have been purified and quantified in milk [154]. A recent groundbreaking study by Gopalakrishna et al. discovered maternal milk to be the principal source of SIgA in the first month of life, when IgA-coating assisted in the reduction of *Enterobacteriaceae* and safeguarded against NEC in mice [155]. This finding was further supported with the observation that IgA-deficient pups exposed to their mother’s milk were still susceptible to NEC [155]. It is noteworthy that γ-*Proteobacteria*-specific IgA is responsible for the transition from the immature to mature microbiota, whereas IgA deficiency results in a bloom of γ-*Proteobacteria* [156]. Recalling that the final transition to a mature microbiota is from γ-*Proteobacteria* to *Clostridia* [151], it can be hypothesized that the *Clostridia* depletion might be an indicator of stalled microbiota maturation in NEC infants. Hence, it would be important for future studies to see whether the supply of maternal IgA correlates with mature microbiota i.e., *Clostridia* restoration in NEC protected infants.

The above studies underscore the therapeutic potential in elevating IgA and modulating the IgA-associated microbiota in NEC. However, targeting IgA in NEC is not necessarily new, as a study back in 1988 by Eibl et al. found oral administration of an IgA-IgG supplement to be effective in preventing NEC in premature infants [157]. Regardless, a therapeutic approach to promote and/or sustain IgA levels could be lifesaving. This is especially important when considering that mothers with inflammatory bowel disease have lower IgA availability to horizontally transfer to infants via breast milk [158] where, potentially, both mother and child could need IgA supplementation. However, awareness must be given to the possibility of IgA ‘over coating’ as a recent study by Brawner et al. described prenatal stress increased IgA coating in the offspring microbiota and aggravated NEC in a sex-dependent manner [159]. As such, other microbial approaches i.e., probiotics can also be used to treat NEC with special regards to *Lactobacillus* spp. [160,161,162]. Prebiotic treatment has not yielded any definitive results but is an interesting area of future research [137].

### 5.3. IgA-Microbiota and Inflammatory Bowel Diseases

Inflammatory bowel diseases (IBD) are the result of intense inflammation throughout the gastrointestinal tract, and there are an estimated 70,000 new cases of IBD diagnosed each year with 6.8 million cases globally [163,164]. It is well established that the microbiota plays an integral part in IBD, but whether its role is the cause, consequence, or a correlation has been a continual question in IBD research. On the one hand, certain bacteria may be the antigenic stimulus responsible for the escalation of inflammatory processes essential in IBD progression. Indeed, GF mice indicated the microbiota as an inducer and aggressor in experimentally-induced and spontaneous colitis, respectively [165]. On the other hand, when studying spontaneous colitis in mice deficient in the epithelium-specific polarized sorting factor adaptor protein (AP)-1B, Jangid et al. found a predisposition to IBD triggered an unfavorable change in the microbiota composition toward dysbiosis, in which a bloom of sulfur-reducing and lactate-producing bacteria might have explained the aggravated colitis [166]. Additional mouse studies support certain individual bacterium as procolitogenic, including the human-derived butyrate-producing strain *Anaerostipes hadrus* BPB5, mucin-eating *Mucispirillum*, *Klebsiella pneumonia*, and *Proteus mirabilis* [167,168,169]. This is further correlated with human studies that observed an increase in the family *Enterobacteriaceae* and the phylum Proteobacteria in IBD patients [170]. These changes have been simultaneously noted as biomarkers for human IBD and a therapeutically related target to abate microbiota from exacerbating IBD. While these studies suggest microbiota composition differences are simply a complication of inflammation, it is noteworthy that a decrease in acetate-butyrate-converting *Roseburia* spp. in healthy controls has been recently found to precede IBD and be sustained during IBD [171], suggesting that microbiota are involved in the etiology of the disease itself.

Given its previously established ability to affect microbiota composition in the gut, SIgA is of much importance in the microbiota-IBD relationship in which IBD patients may have dysfunction in mucosal tolerance to commensal fungi and bacteria. For instance, pIgR knockout mice were more susceptible to colitis due to their defect in SIgA transport and unstable microbiota [172]. Regarding fungi, several *Candida* species are linked with IBD pathology, with particular *Candida* hyphal morphotypes associated with increased IBD severity. Further, SIgA was observed to target adhesion and hyphal cells in pathogenic fungi to prevent *Candida*-associated damage during colitis [103]. Concerning bacteria, mice deficient in inducible costimulator ligand (ICOSL), which are spontaneously susceptible to IBD, have reduced IgA and impaired antigen recognition toward flagellin from mucus-associated bacteria of the *Lachnospiraceae* family [173]. In addition, activating transcription factor 3 (ATF3)-deficient mice exhibit gut microbiota dysbiosis that favors pro-inflammatory *Prevotella copri* abundance, and display impaired T_FH_ cell development in the gut, resulting in significantly decreased SIgA production [174,175]. This response is similar in mice deficient in innate effector protein, myeloid differentiation primary response 88 (MyD88), which plays a key role in modulating IgA responses to the gut microbiota though induction of CD4^+^ T cells and regulatory T cells. In addition, the MyD88 deficient mice displayed exacerbated colitis severity with gut dysbiosis highlighted by an overabundance of SFB and increased bacterial load, indicating MyD88 signaling is needed in IgA responses to IBD and gut dysbiosis [176,177]. Comparatively, deletion of methylation-controlled J protein (a mitochondrial inner membrane protein) caused a bloom in the IBD-associated bacterium *Ruminococcus gnavus*, but surprisingly increased SIgA levels [178]. Likewise, indoleamine 2,3-dioxygenase (IDO) knockout mice had higher basal levels of SIgA reactive toward *Citrobacter rodentium* and were resistant to *Citrobacter*-induced colitis [179]. These last two studies indicate that in certain conditions, elevated SIgA could be a compensatory response to ensure commensal clearance and create an environment ready to fight against pathogens. 

During IBD, SIgA-mediated gut pathogen clearance appears to favor the coating method (see Section 4.2), as a recent study demonstrated IBD patients to have higher amounts of SIgA-coated bacteria in stools compared with controls [180]. As such, IgA-Seq has been utilized to profile the SIgA-coated bacteria and thus identify IBD-associated microbes [181,182,183,184]. For instance, Palm et al. conducted a landmark study by selecting SIgA-coated bacteria via IgA-Seq, isolating and anaerobically culturing these microbes from IBD patients, and then colonizing them in GF mice; finding those SIgA-coated bacteria to be indeed colitogenic [181]. Besides serving as a biomarker, SIgA coating seems to serve as a target for immune-mediated lowering of intestinal bacterial load. A study by Gupta et al. demonstrated this in that a SIgA-high mouse strain CBA/CaJ (CBA) was resistant to dextran sodium sulfate (DSS)-induced acute colitis due to its inherit increased SIgA-coating and decreased fecal bacterial loads, whereas a SIgA-low mouse strain C57BL/6 (B6) was susceptible to colitis [185]. Impressively, a recent report by Rochereau et al. uncovered a subset of Crohn’s disease patients with a mutation in nucleotide binding oligomerization domain containing 2 (NOD2) who had an increase in retrograde transport of antigen-carrying SIgA into the Peyer’s patches [186]. The authors confirmed this observation in NOD2-deficient mice, which supported the concept that increased mucosal inflammation could be due to overactive SIgA retrograde transport [186]. 

Generally, this evidence indicates that a high IgA response in humans may protect against colitis and, therefore, elimination or suppression of SIgA-coated bacteria is an avenue for potential therapies. A study by Zhang et al. found that sodium butyrate treatment to IL-10 deficient mice decreased the number of SIgA-coated bacteria and concomitantly increased gut biodiversity compared to isotype controls [187]. As a more direct approach, Okai et al. recently developed an engineered IgA clone, W27, which targeted and suppressed harmful commensal bacteria, but not beneficial bacteria, resulting in the prevention of colitis and enrichment of gut microbiota diversity in several mouse models [188]. Another source to consider is breast milk-derived SIgA, given that Rogier et al. demonstrated its early exposure ameliorated DSS-induced epithelial damage [189]. It would be interesting to investigate whether this observation could be due to the immunologic tuning of RORγ-expressing regulatory T cells over multi-generational transmission [190]. The continued research in targeting the IgA-microbiota axis could certainly advance the clinical setting for treating IBD.

### 5.4. IgA-Microbiota in Colorectal Cancer 

Colorectal cancer (CRC) is the second most common cause of cancer related deaths in the United States, and IBD happens to be the main precursor for CRC development. In 2021, it is predicted that there will be an estimated 149,500 diagnosed cases and 52,980 deaths from CRC in the United States [191]. As with any cancer, early detection is vital to reduce mortality because treatment can immediately begin after diagnosis. Several studies have investigated autoreactive antibodies with special emphasis on IgA as a prominent CRC screening tool [192,193,194]. In particular, IgA reactive to the tumor associated antigen carcinoembryonic antigen (CEA) has been an important signature for CRC patients. Butviloskaya et al. recently demonstrated with hydrogel biochips that combining anti-CEA and anti-glycan antibodies in diagnosis provided preferable predictive values [195]. Of special note, when immunizing CRC patients with recombinant CEA, IgA anti-CEA antibodies were found to be cytotoxic against the tumor cells and improved patient survival [196]. IgA specific to certain bacteria such as *Fusobacterium nucleatum* and toxin-producing *Clostridioides difficile* has also proven to have diagnostic value with high specificity and sensitivity [197,198,199]. 

While IBD patients suffer from IgA insufficiency, recent literature could suggest that the lack of IgA may actually be a protective mechanism to prevent aggressive CRC. In the *Apc^Min/+^* CRC mouse model, an expansion of IgA^+^ lymphocytes in the tumor microenvironment was identified [200]. This matches the dominancy of plasma cells in advanced tumors for CRC patients, where the B cell subpopulation IgA^+^ IGLC2^+^ was associated with poor prognosis [201]. Noteworthy is the observation that pre-B like cells may have antitumor functions in the early stages of CRC development [201], but perhaps this becomes less effective when they differentiate into plasma cells in advanced CRC. A recent study led by Hale et al. confirmed that a defect in class switching decreased the incidence of inflammation-associated colorectal neoplasia and reduced neoplastic lesions [202]. Specifically, *Tnf*^−/−^ *Il10*^−/−^ (“T/I”) mice prone to spontaneous colitis were bred with *Aicda*^−/−^ (AID encoding gene) mice to generate TIA mice that had no IgA class switching nor somatic hypermutation capabilities. While colitis was still prevalent after 28 weeks of age, the incidence of neoplasia in TIA mice was lowered by nearly 25% when compared to T/I mice [202]. Contrary to the idea of the host purposely limiting IgA availability per se, the reported lack of IgA migration to colonic tumor cells could, theoretically, promote a pro-inflammatory environment that supports oncogenic growth [203]. Mice deficient of IL-33 were shown to have markedly low IgA levels, a dysbiotic microbiota, colitis, and eventual CRC development [204], supporting the conventional concept that IgA is necessary to sustain microbiota homeostasis for the prevention of intestinal diseases. 

The gut microbiome and IgA have a relatively undescribed relationship with CRC, for which future research could unveil new ways to screen and treat this lethal cancer. An interesting research direction would be to understand the role of IgA-coating toward CRC-associated bacteria such as *F. nucleatum* and *B. fragilis* in disease progression [205]. There could be many other gut microbes that may play a role in CRC that require further studies. 

### 5.5. IgA Nephropathy and Vasculitis 

First discovered by Berger and Hinglais in 1968 [206], IgA nephropathy (IgAN, *alias* Berger’s disease) describes the deposition of galactose-deficient IgA1 in the glomerular mesangium and subsequent glomerulonephritis due to the formation of inflammatory immune complexes in the kidney [207,208,209]. IgAN is the most common primary glomerulonephritis worldwide and is often clinically characterized by asymptomatic hematuria and progressive kidney disease [210,211]. Recent estimates suggest approximately one in four patients with IgAN end up developing end-stage renal disease within 20 years and, therefore, have increased risk for mortality [212]. The etiology behind IgAN seems to start with the expansion in intestinal-activated B cells and antibody-secreting cells (ASC) in the lamina propria [213,214]. Both mouse and human studies indicate that the transgenic expression of either APRIL or the high homology BAFF causes aberrant *O*-glycosylation on the IgA1 hinge region [215,216] and the hyperresponsiveness in IgA1 production [217,218,219,220]. When IgA^+^ ASC leave the secondary lymphoid tissue into circulation, they can further differentiate into long-lived IgA^+^ plasma cells. The mesangial deposits of galactose-deficient IgA1 can then over activate the complement system [221,222] and/or complex with IgG autoantibodies [223], which collectively causes proinflammatory responses and renal injury.

Though IgAN is a disease affecting the kidneys, its origins are also heavily linked to a gut microbiota-renal axis. In 2011, Kiryluk et al. found four novel IgAN loci enriched in KEGG pathways associated with the “Intestinal Immune Network for IgA Production”, including a strong positive association to mucosal immunity i.e., local pathogen diversity [224]. More recently, in 2021, He et al. uncovered several microbiome quantitative trait loci associated with the microbial composition changes seen in IgAN e.g., decreased abundances of *Dialister* and *Bacilli* but increased abundances of *Erysipelotrichaceae* and *Lachnobacterium* [225]. Using operational taxonomic units (OTUs), Dong et al. examined for differences in microbial composition between IgAN patients and healthy controls. This study found no gross differences in microbiota diversity or richness, but there were significant taxonomical alterations of key bacteria in IgAN patients including elevated levels of *Escheria-Shigella* and decreased levels of *Roseburia, Lachnospiraceae_*unclassified*, Clostridium_sensu_stricto_1*, and *Fusobacterium* [226]. Interestingly, certain gut metabolites such as short chain fatty acids, in parallel with their bacterial producers, were significantly reduced in IgAN patients [227]. It is notable that antibiotic treatment to humanized mice was sufficient to significantly reduce the pathophysiological features of IgAN, including IgA1 mesangial deposition, immune complexes, and glomerular inflammation [228]. Hence, the gut microbiota appears as a strong contributor to the generation of mucosa-derived nephrotoxic IgA1, but more studies are warranted to further define the gut microbial signatures in IgAN. It is noteworthy that microbial proteases can remove IgA immune complexes from the glomeruli [229], indicating an opportunity to therapeutically resolve IgAN in a microbiota-dependent manner.

Often found concomitantly in IgAN patients is IgA vasculitis (IgAV), alternatively known as Henoch-Schönlein Purpura, a disease in which IgA deposits in blood vessels lead to inflammation. It has been debated as to whether IgAV and IgAN are two clinical manifestations of the same disease in different tissues [230]. The etiological concept of both diseases is essentially the same, but there are minor differences in terms of symptoms and epidemiology. Compared to IgAN occurring primarily in adults and hematuria being the first clinical indicator, IgAV patients are more common in the pediatric population and they exhibit cutaneous (palpable purpura i.e., purple-red rashes), gastrointestinal (colicky pain, bloody stools) and articular (arthralgia i.e., joint pain) symptoms [230]. When comparing gut microbiota profiles, IgAV patients exhibit the marked decrease in diversity seen with IgAN patients, but *Fusobacteria* was found to be increased in IgAV patients [231,232], which is opposite to an early study on IgAN patients. IgAV patients are also noted to have a negative correlation with *Dialister* [231], a previous member of the *Clostridia* family, and a greater abundance of *Escherichia-Shigella* [232], both observations of which parallel IgAN microbiota signatures. It is interesting to note that other cases of vasculitis, such as Kawasaki disease, also exhibit more *Fusobacteria*, whereas Behçet syndrome patients have lower butyrate-producing bacteria, such as *Roseburia* and *Clostridia* spp. [233,234,235,236]. This hints that *Fusobacteria* are most likely pathogenic bacteria in vasculitis, whereas the short chain fatty acid butyrate is perhaps a beneficial metabolite that has limited availability during vasculitis and nephropathy. Nonetheless, larger clinical studies are required to understand the correlation of microbiota in nephropathy and vasculitis, and to also assess microbiota-dependent prognosis and therapeutic strategies for IgAN and IgAV.

### 5.6. IgA-Microbiota in Salmonella Infection

Salmonellosis is a food borne diarrheal disease that presents itself as either gastroenteritis or enterocolitis. Most *Salmonella* spp., except for *S. typhi* and *S. paratyphi*, can infect the gastrointestinal tract, but *Salmonella enterica* strains are the most prevalent. *Salmonella* infection is a global concern, in which approximately 20 million people yearly contract the bacterium and there are more than 200,000 deaths annually [237]. In the United States alone, there is an average of over 38,000 cases reported to the Center for Diseases Control, and the highest incidence is among children under 5 years old with a rate of 45 cases/100,000 population [238]. *Salmonella enterica* serovars Typhimurium (STm) is the primary infection model discussed in this review. 

Invasive and noninvasive STm have a type III secretion system that facilities docking and invasion of the intestinal epithelium by the ejection of bacterial toxins [239]. Invasive STm infects the intestinal epithelia by exclusive entry into M cells within the follicle-associated epithelium of the Peyer’s patches [240]. Noninvasive STm, on the other hand, can be captured by DCs in the lamina propria independent of M cells. The invasive STm in the Peyer’s patches stimulates IgA responses, whereas noninvasive STm does not induce IgA responses in the intestinal epithelium but enters into the lamina propria then disseminates into the mesenteric lymph nodes via CCR7, continues to the bloodstream and spleen via CD18-expressing phagocytes, and lastly triggers humoral IgG responses [241,242,243]. Besides the resident DCs, other subsets of CXCR3^+^ mononuclear phagocytes, such as CXCL13^+^ macrophages, function as superior mucosal antigen-presenting cells for the recruitment and activation of CD4^+^ T cells and IgA-producing B cells [34]. The CXCR3^+^ cells can additionally participate in immune exclusion in the early stage of infection by migrating to the intestinal lumen to capture bacteria [244]. Accordingly, the absence of CXCR3 results in greater *Salmonella* load due to the compromised mucosal IgA responses [245]. In line with this, the absence of Peyer’s patches in mice causes a failure of *Salmonella*-specific intestinal IgA production [246,247].

Understanding the mechanisms concerning how IgA fends off *Salmonella* has been elucidated mostly from studies that utilized recombinant IgA monoclonal antibodies. Generation of the O5 serotype-specific IgA monoclonal antibody, called Sal4, was derived after the first observation that mice bearing subcutaneous Sal4 hybridoma tumors were protected from an oral challenge of STm [102]. Interestingly, Sal4 in dimeric and SIgA forms, but not recombinant IgG1 carrying the Sal4 variable regions, were effective to prophylactically immunize mice and act as a curative treatment against STm [248]. Upon oral administration, Sal4-SIgA promotes bacterial agglutination via cross-linking, and the clumps of microbes become susceptible to either immune exclusion or enchained growth for clearance, depending on the bacterial density [104,249,250]. Similar to Sal4, a biologically active secretory-like IgA and IgM (SCIgA/M) antibody protected mice from intragastric infection with a virulent strain of STm as revealed by reduced colonization of both mucosal and systemic compartments, and preserved integrity of the Peyer’s patches through immune exclusion of bacterial aggregates [250,251]. While these recombinant monoclonal antibodies signify the bactericidal ability of SIgA, there are conflicting reports as to whether innate SIgA confers protection against STm. In two independent reports, pIgR-deficient mice were infected with STm SL1344 at either a dose of 10^9^ CFU or 10^7^ CFU, respectively, where the first group demonstrated increased survival and protection from systemic dissemination of STm, but the second group had more profound infection [252,253]. It is noteworthy, though, that previously immunized pIgR-deficient mice and their wild-type litter mates were equally resistant to STm challenge [254]. These collectively indicate that immunization against STm is one of the best prophylactic defense options. While no vaccine has been approved yet, an exciting recent 2021 report from Zhao et al. constructed and tested a live attenuated vaccine strain of STm and found it to exhibit exceptional immunogenicity and protection efficacy in mice prior to STm challenge [255].

There are limited studies that have investigated the relationship of the IgA-microbiota axis in *Salmonella* infection. A study by Endt et al. described the contributions between SIgA and microbiota in mucosal defense and STm clearance, respectively. By using a low complex microbiota-derived mouse model, they found that SIgA functions to restrict pathogen access to the mucosal surface but played no part in the kinetics of bacterial clearance [256]. The introduction of a complex microbiota to the mouse model mediated STm clearance independent of Th_17_ and SIgA [256], and the mechanism to how is still unknown. Futures studies are certainly needed to further define the interaction between SIgA and microbiota in STm pathogenesis and clearance.

### 5.7. IgA-Microbiota in Biliary Infection

Bile is a greenish-yellow secretory product responsible for the emulsification of lipids and fat-soluble vitamins from our diet. The liver is responsible for bile synthesis and its transportation into the gallbladder for storage. In humans, roughly 5–50 µg/mL of protein is excreted from the liver in the form of bile per day [257]. Ig are predominant biliary proteins with IgG prevailing in hepatic bile and IgA most abundant in gallbladder bile [258]. Specifically, gallbladder bile contains a pool of polymeric IgA, polymeric SIgA, and free secretory component (SC). The mechanisms for IgA transportation into the gallbladder vary among species. In rats, for instance, circulating polymeric IgA binds to the SC expressed on hepatocyte sinusoidal plasma membranes and then internalizes into endocytic vesicles as SIgA. Next, the vesicles travel to the bile canalicular membrane where pIgR expressed on biliary epithelial cells promotes transcytosis of SIgA into bile [259,260,261]. In humans, polymeric IgA is produced by adjacent plasma cells along the hepatobiliary tree and is then captured by the SC-pIgR complex expressed on biliary epithelial cells for secretion into bile [258,260,261]. This difference between capturing IgA transport and secretion into bile between rats and humans, respectively, is due to the presence of SC on hepatocytes in rats but not in humans, who express SC only on the biliary epithelium [260,262]. 

The large presence of SIgA in the bile suggests an important biological function of IgA within the hepatobiliary system. There are several proposed functions for IgA in bile [259]. Much research from the 1980s concluded that IgA transport from circulation to bile serves as a natural route to remove antigens [263,264]. Radiolabeled antigens intravenously injected with various immunoglobulin classes into mice showed IgA, but not IgG nor IgM, to be the primary antibody for antigen transport into bile [264]. This function of bile IgA is essential for abating hepatobiliary infections that are secondary consequences from intestinal bacterial and parasitic infections, in addition to preventing primary liver infections [265,266,267,268,269,270]. By immunizing rats, via injection of a killed *E. coli* strain into Peyer’s patches, the production of biliary IgA-specific anti-*E. coli* protected against hepatobiliary infection, cholangitis, and systemic sepsis [271]. It must be cautioned that patients with hepatobiliary diseases (e.g., cholestasis, cholelithiasis) are at greater risk for gallbladder infection because the injury to biliary epithelial cells results in impaired hepatobiliary IgA clearance and bile IgA reflux into the blood [261,272]. In line with this, individuals with SIgAD are naturally more prone to hepatobiliary diseases such as primary biliary cirrhosis and gallbladder infections [273,274]. It is interesting that a test for IgA-coated bacteria in the bile fluid can relate to clinical symptoms such as fever and leukocytosis for those with hepatobiliary infection [275].

Not much research has investigated whether the gut microbiota could influence IgA in hepatobiliary infections. Only recently, Moro-Sibilot et al. in 2016 demonstrate that local IgA in the liver was identified to be microbiota reactive and sourced from antibody secreting cells (i.e., plasmablasts) that left the Peyer’s patches [276]. Prior research has also indicated that patients with gallstones exhibit gut microbiota dysbiosis, and that about 70% of gut bacterial OTUs (OTU) were detectable in the biliary tract [277]; however, whether that impacts liver and bile IgA is unknown. Recently, more recognition has been given to changes in the bile microbiota with biliary infection. In parallel with the gut microbiota, the four dominant phyla are *Proteobacteria*, *Firmicutes*, *Bacteroidetes* and *Actinobacteria* in the bile microbiota [278]. The presence of liver fluke *Opisthorchis felineus* infection resulted in an increase of beta-diversity for the bile microbial community with members of the *Spirochaetes* phylum blooming and elevated abundance of *Klebsiella* spp., *Aggregatibacter* spp., *Lactobacillus* spp., *Treponema* spp., *Haemophilus parainfluenzae* and *Staphylococcus equorum* [278,279]. Another important finding was that the infected individuals had detectable levels of *Veillonella dispar*, *Paracoccus aminovorans*, *Parabacteroides distasonis*, *Sphingomonas changbaiensis*, *Cellulosimicrobium* spp., and *Phycicoccus* spp. that were not found in the uninfected patients [278]. Changes in the gut microbiota are also noted following liver fluke infection, such as increases in *Lachnospiraceae*, *Ruminococcaceae*, and *Lactobacillaceae*, but a decrease *Porphyromonadaceae*, *Erysipelotrichaceae*, and *Eubacteriaceae* [280]. Future studies should determine whether single or concurrent changes between the biliary and gut microbiota impact IgA function and whether this could determine severity of hepatobiliary infection.

**Table 1:** IgA Involvement in Various Diseases.

Disease	Involvement of IgA
Autoimmune Disease [Section 5.1]	Selective IgA deficiency has a two-hit impact causing pathobiont expansion leading to dysbiosis and spontaneous gut inflammation [122,127] and unmitigated inflammation leading to autoimmune disease development [120].
Necrotizing Enterocolitis (NEC) [Section 5.2]	Dimeric IgA in mother’s milk helps to control the prevalence of *Enterobacteriaceae* providing a safeguard against NEC [151].
Inflammatory Bowel Diseases (IBD) [Section 5.3]	SIgA aids in preventing IBD pathogenesis by helping to facilitate microbiota stability [172], neutralization of procolitogenic fungi and bacteria via immune exclusion [103,180].
Colorectal Cancer (CRC) [Section 5.4]	IgA antibodies reactive to carcinoembryonic antigen to be cytotoxic to colonic tumor cells [196]. Promotes a proinflammatory tumor microenvironment for oncogenic growth [203].
Nephropathy & Vasculitis [Section 5.5]	IgA aggregates in the glomerulus of the kidney causing inflammation leading to nephropathy [198,199,200]. IgA deposits in the walls of the blood vessel leading to vasculitis [221].
*Salmonella* Infection [Section 5.6]	Promotes bacterial agglutination to become susceptible to immune exclusion or enchained growth by SigA for clearance [104,249,250].
Biliary Infection [Section 5.7]	SIgA is the predominant antibody in bile and helps to prevent primary and secondary hepatobiliary infection from intestinal or parasitic infection [258,265,266,267,268,269,270].

## 6. Therapeutic Potential of IgA

From a microbiological viewpoint, key questions remain concerning how to target IgA. From its unique functional capabilities, it is necessary to consider the clinical applications of IgA. To date, very few therapeutic IgA treatments have been approved for use in the United States. Immunoglobulin replacement therapy using antibodies purified from donated plasma has been practiced as a conventional treatment for IgA deficiencies [281]. Novel therapies including synthetically engineered multivalent bispecific antibodies (BsAbs) have been developed and are clinically approved to treat various cancers such as acute lymphoblastic leukemia [282] and small cell lung cancer [283], but currently no BsAbs have been approved and only limited to treat IgA-related disorders [284,285]. Positive reports in rodent models support BsAbs to be effective in alleviating IBD [286,287]. Certain commensals from the microbiota (e.g., *Lactobacillus lactis*) have been demonstrated as a delivery system to secrete these BsAbs for the improvement of IgA-associated diseases such as colitis [288]. It can be expected that IgA-based BsAbs, in conjunction with the frontline microbiota target therapeutics i.e., probiotics and prebiotics, would underpin a paradigm shift in the treatment of immunological disorders and communicable diseases. 

## 7. Future Thoughts

Compelling evidence demonstrates IgA to be the fundamental immunoglobulin that bridges the host and microbiota into symbiosis. As IgA-microbiota interactions are extremely diverse and complex across bacterial and fungal species, more studies are needed to delineate the specific mechanisms by which IgA binding modifies physiology and/or fitness of the microbial community. It is understood that IgA has multiple methods to facilitate appropriate antigenic specificity and immune responses, but whether antibody coating could cause unintended consequences for the microbiota status is of future concern. Dysfunctions in IgA, such as abnormal glycosylation, receptor shedding, and self-aggregation, result in inflammatory diseases that can affect a wide array of organ systems. With emerging reports regarding dietary components as other antigens recognized by SIgA, future studies are warranted to delineate the complex interaction among diet, microbiota, and SIgA in both physiological and pathophysiological conditions. The answers to these queries would help better interpret the immunological functions of IgA and have significant implications in translational therapeutics that target the IgA-microbiota axis. 

## References

[1] Gutzeit C., Magri G., Cerutti A. (2014). Intestinal IgA production and its role in host-microbe interaction. Immunol. Rev..

[2] Weis A.M., Round J.L. (2021). Microbiota-antibody interactions that regulate gut homeostasis. Cell Host Microbe..

[3] Mkaddem S.B., Christou I., Rossato E., Berthelot L., Lehuen A., Monteiro R.C., Daeron M., Nimmerjahn F. (2014). IgA, IgA Receptors, and Their Anti-inflammatory Properties. Fc Receptors.

[4] Petersen C., Bell R., Klag K.A., Lee S.H., Soto R., Ghazaryan A., Buhrke K., Ekiz H.A., Ost K.S., Boudina S. (2019). T cell-mediated regulation of the microbiota protects against obesity. Science.

[5] Abdel-Gadir A., Stephen-Victor E., Gerber G.K., Noval Rivas M., Wang S., Harb H., Wang L., Li N., Crestani E., Spielman S. (2019). Microbiota therapy acts via a regulatory T cell MyD88/RORgammat pathway to suppress food allergy. Nat. Med..

[6] Stanfield R.L., Wilson I.A. (2014). Antibody Structure. Microbiol. Spectr..

[7] Schroeder H.W., Cavacini L. (2010). Structure and function of immunoglobulins. J. Allergy Clin. Immunol..

[8] Gabrielli E., Pericolini E., Cenci E., Ortelli F., Magliani W., Ciociola T., Bistoni F., Conti S., Vecchiarelli A., Polonelli L. (2009). Antibody complementarity-determining regions (CDRs): A bridge between adaptive and innate immunity. PLoS ONE.

[9] Kishikawa S., Sato S., Kaneto S., Uchino S., Kohsaka S., Nakamura S., Kiyono H. (2017). Allograft inflammatory factor 1 is a regulator of transcytosis in M cells. Nat. Commun..

[10] Rios D., Wood M.B., Li J., Chassaing B., Gewirtz A.T., Williams I.R. (2016). Antigen sampling by intestinal M cells is the principal pathway initiating mucosal IgA production to commensal enteric bacteria. Mucosal. Immunol..

[11] Neutra M.R. (1998). Current concepts in mucosal immunity. V Role of M cells in transepithelial transport of antigens and pathogens to the mucosal immune system. Am. J. Physiol..

[12] Lelouard H., Fallet M., de Bovis B., Meresse S., Gorvel J.P. (2012). Peyer’s patch dendritic cells sample antigens by extending dendrites through M cell-specific transcellular pores. Gastroenterology.

[13] Lelouard H., Henri S., De Bovis B., Mugnier B., Chollat-Namy A., Malissen B., Meresse S., Gorvel J.P. (2010). Pathogenic bacteria and dead cells are internalized by a unique subset of Peyer’s patch dendritic cells that express lysozyme. Gastroenterology.

[14] Reboldi A., Arnon T.I., Rodda L.B., Atakilit A., Sheppard D., Cyster J.G. (2016). IgA production requires B cell interaction with subepithelial dendritic cells in Peyer’s patches. Science.

[15] Qi H., Egen J.G., Huang A.Y., Germain R.N. (2006). Extrafollicular activation of lymph node B cells by antigen-bearing dendritic cells. Science.

[16] Borsutzky S., Cazac B.B., Roes J., Guzman C.A. (2004). TGF-beta receptor signaling is critical for mucosal IgA responses. J. Immunol..

[17] Cerutti A. (2008). The regulation of IgA class switching. Nat. Rev. Immunol..

[18] Nakamura Y., Mimuro H., Kunisawa J., Furusawa Y., Takahashi D., Fujimura Y., Kaisho T., Kiyono H., Hase K. (2020). Microfold cell-dependent antigen transport alleviates infectious colitis by inducing antigen-specific cellular immunity. Mucosal. Immunol..

[19] Salazar-Gonzalez R.M., Niess J.H., Zammit D.J., Ravindran R., Srinivasan A., Maxwell J.R., Stoklasek T., Yadav R., Williams I.R., Gu X. (2006). CCR6-mediated dendritic cell activation of pathogen-specific T cells in Peyer’s patches. Immunity.

[20] Lycke N.Y., Bemark M. (2017). The regulation of gut mucosal IgA B-cell responses: Recent developments. Mucosal. Immunol..

[21] Hirota K., Turner J.E., Villa M., Duarte J.H., Demengeot J., Steinmetz O.M., Stockinger B. (2013). Plasticity of Th17 cells in Peyer’s patches is responsible for the induction of T cell-dependent IgA responses. Nat. Immunol..

[22] Tsuji M., Komatsu N., Kawamoto S., Suzuki K., Kanagawa O., Honjo T., Hori S., Fagarasan S. (2009). Preferential generation of follicular B helper T cells from Foxp3+ T cells in gut Peyer’s patches. Science.

[23] Martinez-Lopez M., Iborra S., Conde-Garrosa R., Mastrangelo A., Danne C., Mann E.R., Reid D.M., Gaboriau-Routhiau V., Chaparro M., Lorenzo M.P. (2019). Microbiota Sensing by Mincle-Syk Axis in Dendritic Cells Regulates Interleukin-17 and -22 Production and Promotes Intestinal Barrier Integrity. Immunity.

[24] Crotty S. (2014). T follicular helper cell differentiation, function, and roles in disease. Immunity.

[25] Huus K.E., Petersen C., Finlay B.B. (2021). Diversity and dynamism of IgA−microbiota interactions. Nature Rev. Immunol..

[26] Gohda M., Kunisawa J., Miura F., Kagiyama Y., Kurashima Y., Higuchi M., Ishikawa I., Ogahara I., Kiyono H. (2008). Sphingosine 1-phosphate regulates the egress of IgA plasmablasts from Peyer’s patches for intestinal IgA responses. J. Immunol..

[27] Mora J.R., Iwata M., Eksteen B., Song S.Y., Junt T., Senman B., Otipoby K.L., Yokota A., Takeuchi H., Ricciardi-Castagnoli P. (2006). Generation of gut-homing IgA-secreting B cells by intestinal dendritic cells. Science.

[28] Kawamoto S., Tran T.H., Maruya M., Suzuki K., Doi Y., Tsutsui Y., Kato L.M., Fagarasan S. (2012). The inhibitory receptor PD-1 regulates IgA selection and bacterial composition in the gut. Science.

[29] Perruzza L., Strati F., Gargari G., D’Erchia A.M., Fosso B., Pesole G., Guglielmetti S., Grassi F. (2019). Enrichment of intestinal Lactobacillus by enhanced secretory IgA coating alters glucose homeostasis in P2rx7(-/-) mice. Sci. Rep..

[30] Perruzza L., Gargari G., Proietti M., Fosso B., D’Erchia A.M., Faliti C.E., Rezzonico-Jost T., Scribano D., Mauri L., Colombo D. (2017). T Follicular Helper Cells Promote a Beneficial Gut Ecosystem for Host Metabolic Homeostasis by Sensing Microbiota-Derived Extracellular ATP. Cell Rep..

[31] Melo-Gonzalez F., Kammoun H., Evren E., Dutton E.E., Papadopoulou M., Bradford B.M., Tanes C., Fardus-Reid F., Swann J.R., Bittinger K. (2019). Antigen-presenting ILC3 regulate T cell-dependent IgA responses to colonic mucosal bacteria. J. Exp. Med..

[32] McCarron M.J., Marie J.C. (2014). TGF-beta prevents T follicular helper cell accumulation and B cell autoreactivity. J. Clin. Investig..

[33] Jung Y., Wen T., Mingler M.K., Caldwell J.M., Wang Y.H., Chaplin D.D., Lee E.H., Jang M.H., Woo S.Y., Seoh J.Y. (2015). IL-1beta in eosinophil-mediated small intestinal homeostasis and IgA production. Mucosal. Immunol..

[34] Koscso B., Kurapati S., Rodrigues R.R., Nedjic J., Gowda K., Shin C., Soni C., Ashraf A.Z., Purushothaman I., Palisoc M. (2020). Gut-resident CX3CR1(hi) macrophages induce tertiary lymphoid structures and IgA response in situ. Sci. Immunol..

[35] Litinskiy M.B., Nardelli B., Hilbert D.M., He B., Schaffer A., Casali P., Cerutti A. (2002). DCs induce CD40-independent immunoglobulin class switching through BLyS and APRIL. Nat. Immunol..

[36] Yu G., Boone T., Delaney J., Hawkins N., Kelley M., Ramakrishnan M., McCabe S., Qiu W.R., Kornuc M., Xia X.Z. (2000). APRIL and TALL-I and receptors BCMA and TACI: System for regulating humoral immunity. Nat. Immunol..

[37] Castigli E., Wilson S.A., Scott S., Dedeoglu F., Xu S., Lam K.P., Bram R.J., Jabara H., Geha R.S. (2005). TACI and BAFF-R mediate isotype switching in B cells. J. Exp. Med..

[38] Castigli E., Scott S., Dedeoglu F., Bryce P., Jabara H., Bhan A.K., Mizoguchi E., Geha R.S. (2004). Impaired IgA class switching in APRIL-deficient mice. Proc. Natl. Acad. Sci. USA.

[39] Matsuo K., Nagakubo D., Yamamoto S., Shigeta A., Tomida S., Fujita M., Hirata T., Tsunoda I., Nakayama T., Yoshie O. (2018). CCL28-Deficient Mice Have Reduced IgA Antibody-Secreting Cells and an Altered Microbiota in the Colon. J. Immunol..

[40] Grootjans J., Krupka N., Hosomi S., Matute J.D., Hanley T., Saveljeva S., Gensollen T., Heijmans J., Li H., Limenitakis J.P. (2019). Epithelial endoplasmic reticulum stress orchestrates a protective IgA response. Science.

[41] Macpherson A.J., Uhr T. (2004). Induction of protective IgA by intestinal dendritic cells carrying commensal bacteria. Science.

[42] McDole J.R., Wheeler L.W., McDonald K.G., Wang B., Konjufca V., Knoop K.A., Newberry R.D., Miller M.J. (2012). Goblet cells deliver luminal antigen to CD103+ dendritic cells in the small intestine. Nature.

[43] Tezuka H., Abe Y., Iwata M., Takeuchi H., Ishikawa H., Matsushita M., Shiohara T., Akira S., Ohteki T. (2007). Regulation of IgA production by naturally occurring TNF/iNOS-producing dendritic cells. Nature.

[44] Fritz J.H., Rojas O.L., Simard N., McCarthy D.D., Hapfelmeier S., Rubino S., Robertson S.J., Larijani M., Gosselin J., Ivanov I.I. (2011). Acquisition of a multifunctional IgA+ plasma cell phenotype in the gut. Nature.

[45] Bergqvist P., Stensson A., Lycke N.Y., Bemark M. (2010). T cell-independent IgA class switch recombination is restricted to the GALT and occurs prior to manifest germinal center formation. J. Immunol..

[46] Cao A.T., Yao S., Gong B., Nurieva R.I., Elson C.O., Cong Y. (2015). Interleukin (IL)-21 promotes intestinal IgA response to microbiota. Mucosal. Immunol..

[47] Bang Y.-J., Hu Z., Li Y., Gattu S., Ruhn Kelly A., Raj P., Herz J., Hooper Lora V. (2021). Serum amyloid A delivers retinol to intestinal myeloid cells to promote adaptive immunity. Science.

[48] Kumar Bharathkar S., Parker B.W., Malyutin A.G., Haloi N., Huey-Tubman K.E., Tajkhorshid E., Stadtmueller B.M. (2020). The structures of secretory and dimeric immunoglobulin A. Elife.

[49] Steffen U., Koeleman C.A., Sokolova M.V., Bang H., Kleyer A., Rech J., Unterweger H., Schicht M., Garreis F., Hahn J. (2020). IgA subclasses have different effector functions associated with distinct glycosylation profiles. Nat. Commun..

[50] Woof J.M., Russell M.W. (2011). Structure and function relationships in IgA. Mucosal. Immunol..

[51] Phillips-Quagliata J.M. (2002). Mouse IgA allotypes have major differences in their hinge regions. Immunogenetics.

[52] Senior B.W., Dunlop J.I., Batten M.R., Kilian M., Woof J.M. (2000). Cleavage of a recombinant human immunoglobulin A2 (IgA2)-IgA1 hybrid antibody by certain bacterial IgA1 proteases. Infect. Immun..

[53] He B., Xu W., Santini P.A., Polydorides A.D., Chiu A., Estrella J., Shan M., Chadburn A., Villanacci V., Plebani A. (2007). Intestinal bacteria trigger T cell-independent immunoglobulin A(2) class switching by inducing epithelial-cell secretion of the cytokine APRIL. Immunity.

[54] Johansen F.E., Braathen R., Brandtzaeg P. (2000). Role of J chain in secretory immunoglobulin formation. Scand. J. Immunol..

[55] Johansen F.E., Braathen R., Brandtzaeg P. (2001). The J chain is essential for polymeric Ig receptor-mediated epithelial transport of IgA. J. Immunol..

[56] Song W., Bomsel M., Casanova J., Vaerman J.P., Mostov K. (1994). Stimulation of transcytosis of the polymeric immunoglobulin receptor by dimeric IgA. Proc. Natl. Acad. Sci. USA.

[57] Stadtmueller B.M., Huey-Tubman K.E., Lopez C.J., Yang Z., Hubbell W.L., Bjorkman P.J. (2016). The structure and dynamics of secretory component and its interactions with polymeric immunoglobulins. Elife.

[58] Patel A., Jialal I. (2021). Biochemistry, Immunoglobulin A.

[59] Xiong E., Li Y., Min Q., Cui C., Liu J., Hong R., Lai N., Wang Y., Sun J., Matsumoto R. (2019). MZB1 promotes the secretion of J-chain-containing dimeric IgA and is critical for the suppression of gut inflammation. Proc. Natl. Acad. Sci. USA.

[60] Crottet P., Corthesy B. (1998). Secretory component delays the conversion of secretory IgA into antigen-binding competent F(ab’)2: A possible implication for mucosal defense. J. Immunol..

[61] Renegar K.B., Jackson G.D., Mestecky J. (1998). In vitro comparison of the biologic activities of monoclonal monomeric IgA, polymeric IgA, and secretory IgA. J. Immunol..

[62] Phalipon A., Cardona A., Kraehenbuhl J.P., Edelman L., Sansonetti P.J., Corthesy B. (2002). Secretory component: A new role in secretory IgA-mediated immune exclusion in vivo. Immunity.

[63] Mestas J., Hughes C.C. (2004). Of mice and not men: Differences between mouse and human immunology. J. Immunol..

[64] Gibbons D.L., Spencer J. (2011). Mouse and human intestinal immunity: Same ballpark, different players; different rules, same score. Mucosal. Immunol..

[65] Peng S.L. (2005). Signaling in B cells via Toll-like receptors. Curr. Opin. Immunol..

[66] Nikolova E.B., Russell M.W. (1995). Dual function of human IgA antibodies: Inhibition of phagocytosis in circulating neutrophils and enhancement of responses in IL-8-stimulated cells. J. Leukoc. Biol..

[67] Russell M.W., Reinholdt J., Kilian M. (1989). Anti-inflammatory activity of human IgA antibodies and their Fab alpha fragments: Inhibition of IgG-mediated complement activation. Eur. J. Immunol..

[68] Blank U., Launay P., Benhamou M., Monteiro R.C. (2009). Inhibitory ITAMs as novel regulators of immunity. Immunol. Rev..

[69] Bakema J.E., van Egmond M. (2011). The human immunoglobulin A Fc receptor FcalphaRI: A multifaceted regulator of mucosal immunity. Mucosal. Immunol..

[70] Pasquier B., Launay P., Kanamaru Y., Moura I.C., Pfirsch S., Ruffie C., Henin D., Benhamou M., Pretolani M., Blank U. (2005). Identification of FcalphaRI as an inhibitory receptor that controls inflammation: Dual role of FcRgamma ITAM. Immunity.

[71] Wolf H.M., Fischer M.B., Puhringer H., Samstag A., Vogel E., Eibl M.M. (1994). Human serum IgA downregulates the release of inflammatory cytokines (tumor necrosis factor-alpha, interleukin-6) in human monocytes. Blood.

[72] Rogier E.W., Frantz A.L., Bruno M.E., Kaetzel C.S. (2014). Secretory IgA is Concentrated in the Outer Layer of Colonic Mucus along with Gut Bacteria. Pathogens.

[73] Kurita N., Honda S., Shibuya A. (2015). Increased serum IgA in Fcalpha/muR-deficient mice on the (129 x C57BL/6) F1 genetic background. Mol. Immunol..

[74] Vidarsson G., van Der Pol W.L., van Den Elsen J.M., Vile H., Jansen M., Duijs J., Morton H.C., Boel E., Daha M.R., Corthesy B. (2001). Activity of human IgG and IgA subclasses in immune defense against Neisseria meningitidis serogroup B. J. Immunol..

[75] Lang M.L., Shen L., Gao H., Cusack W.F., Lang G.A., Wade W.F. (2001). Fc alpha receptor cross-linking causes translocation of phosphatidylinositol-dependent protein kinase 1 and protein kinase B alpha to MHC class II peptide-loading-like compartments. J. Immunol..

[76] Park R.K., Izadi K.D., Deo Y.M., Durden D.L. (1999). Role of Src in the modulation of multiple adaptor proteins in FcalphaRI oxidant signaling. Blood.

[77] Gulle H., Samstag A., Eibl M.M., Wolf H.M. (1998). Physical and functional association of Fc alpha R with protein tyrosine kinase Lyn. Blood.

[78] Van Gool M.M.J., van Egmond M. (2020). IgA and FcalphaRI: Versatile Players in Homeostasis, Infection, and Autoimmunity. Immunotargets Ther.

[79] Van der Steen L., Tuk C.W., Bakema J.E., Kooij G., Reijerkerk A., Vidarsson G., Bouma G., Kraal G., de Vries H.E., Beelen R.H. (2009). Immunoglobulin A: Fc(alpha)RI interactions induce neutrophil migration through release of leukotriene B4. Gastroenterology.

[80] Hansen I.S., Hoepel W., Zaat S.A.J., Baeten D.L.P., den Dunnen J. (2017). Serum IgA Immune Complexes Promote Proinflammatory Cytokine Production by Human Macrophages, Monocytes, and Kupffer Cells through FcalphaRI-TLR Cross-Talk. J. Immunol..

[81] Bäckhed F., Ley R.E., Sonnenburg J.L., Peterson D.A., Gordon J.I. (2005). Host-Bacterial Mutualism in the Human Intestine. Science.

[82] Eckburg P.B., Bik E.M., Bernstein C.N., Purdom E., Dethlefsen L., Sargent M., Gill S.R., Nelson K.E., Relman D.A. (2005). Diversity of the human intestinal microbial flora. Science.

[83] Janzon A., Goodrich J.K., Koren O., Group T.S., Waters J.L., Ley R.E. (2019). Interactions between the Gut Microbiome and Mucosal Immunoglobulins A, M, and G in the Developing Infant Gut. mSystems.

[84] Gensollen T., Iyer S.S., Kasper D.L., Blumberg R.S. (2016). How colonization by microbiota in early life shapes the immune system. Science.

[85] Sutherland D.B., Suzuki K., Fagarasan S. (2016). Fostering of advanced mutualism with gut microbiota by Immunoglobulin A. Immunol. Rev..

[86] Tsuruta T., Inoue R., Nojima I., Tsukahara T., Hara H., Yajima T. (2009). The amount of secreted IgA may not determine the secretory IgA coating ratio of gastrointestinal bacteria. FEMS Immunol. Med. Microbiol..

[87] Kabbert J., Benckert J., Rollenske T., Hitch T.C.A., Clavel T., Cerovic V., Wardemann H., Pabst O. (2020). High microbiota reactivity of adult human intestinal IgA requires somatic mutations. J. Exp. Med..

[88] Bunker J.J., Erickson S.A., Flynn T.M., Henry C., Koval J.C., Meisel M., Jabri B., Antonopoulos D.A., Wilson P.C., Bendelac A. (2017). Natural polyreactive IgA antibodies coat the intestinal microbiota. Science.

[89] Benckert J., Schmolka N., Kreschel C., Zoller M.J., Sturm A., Wiedenmann B., Wardemann H. (2011). The majority of intestinal IgA+ and IgG+ plasmablasts in the human gut are antigen-specific. J. Clin. Investig..

[90] Fransen F., Zagato E., Mazzini E., Fosso B., Manzari C., El Aidy S., Chiavelli A., D’Erchia A.M., Sethi M.K., Pabst O. (2015). BALB/c and C57BL/6 Mice Differ in Polyreactive IgA Abundance, which Impacts the Generation of Antigen-Specific IgA and Microbiota Diversity. Immunity.

[91] Sterlin D., Fadlallah J., Adams O., Fieschi C., Parizot C., Dorgham K., Rajkumar A., Autaa G., El-Kafsi H., Charuel J.L. (2020). Human IgA binds a diverse array of commensal bacteria. J. Exp. Med..

[92] Joglekar P., Ding H., Canales-Herrerias P., Pasricha P.J., Sonnenburg J.L., Peterson D.A. (2019). Intestinal IgA Regulates Expression of a Fructan Polysaccharide Utilization Locus in Colonizing Gut Commensal Bacteroides thetaiotaomicron. mBio.

[93] Hapfelmeier S., Lawson M.A., Slack E., Kirundi J.K., Stoel M., Heikenwalder M., Cahenzli J., Velykoredko Y., Balmer M.L., Endt K. (2010). Reversible microbial colonization of germ-free mice reveals the dynamics of IgA immune responses. Science.

[94] Grosserichter-Wagener C., Radjabzadeh D., van der Weide H., Smit K.N., Kraaij R., Hays J.P., van Zelm M.C. (2019). Differences in Systemic IgA Reactivity and Circulating Th Subsets in Healthy Volunteers with Specific Microbiota Enterotypes. Front. Immunol..

[95] Yang C., Mogno I., Contijoch E.J., Borgerding J.N., Aggarwala V., Li Z., Siu S., Grasset E.K., Helmus D.S., Dubinsky M.C. (2020). Fecal IgA Levels Are Determined by Strain-Level Differences in Bacteroides ovatus and Are Modifiable by Gut Microbiota Manipulation. Cell Host Microbe..

[96] Yasui H., Nagaoka N., Mike A., Hayakawa K., Ohwaki M. (1992). Detection of Bifidobacterium Strains that Induce Large Quantities of IgA. Microb. Ecol. Health Dis..

[97] Peterson D.A., McNulty N.P., Guruge J.L., Gordon J.I. (2007). IgA response to symbiotic bacteria as a mediator of gut homeostasis. Cell Host Microbe.

[98] Michetti P., Porta N., Mahan M.J., Slauch J.M., Mekalanos J.J., Blum A.L., Kraehenbuhl J.P., Neutra M.R. (1994). Monoclonal immunoglobulin A prevents adherence and invasion of polarized epithelial cell monolayers by Salmonella typhimurium. Gastroenterology.

[99] Yang Y., Palm N.W. (2020). Immunoglobulin A and the microbiome. Curr. Opin. Microbiol..

[100] Hand T.W., Reboldi A. (2021). Production and Function of Immunoglobulin A. Ann. Rev. Immunol..

[101] Mantis N.J., Rol N., Corthésy B. (2011). Secretory IgA’s complex roles in immunity and mucosal homeostasis in the gut. Mucosal. Immunol..

[102] Michetti P., Mahan M.J., Slauch J.M., Mekalanos J.J., Neutra M.R. (1992). Monoclonal secretory immunoglobulin A protects mice against oral challenge with the invasive pathogen Salmonella typhimurium. Infect. Immunol..

[103] Ost K.S., O’Meara T.R., Stephens W.Z., Chiaro T., Zhou H., Penman J., Bell R., Catanzaro J.R., Song D., Singh S. (2021). Adaptive immunity induces mutualism between commensal eukaryotes. Nature.

[104] Moor K., Diard M., Sellin M.E., Felmy B., Wotzka S.Y., Toska A., Bakkeren E., Arnoldini M., Bansept F., Co A.D. (2017). High-avidity IgA protects the intestine by enchaining growing bacteria. Nature.

[105] Bansept F., Marrec L., Bitbol A.F., Loverdo C. (2019). Antibody-mediated crosslinking of gut bacteria hinders the spread of antibiotic resistance. Evolution.

[106] Bansept F., Schumann-Moor K., Diard M., Hardt W.D., Slack E., Loverdo C. (2019). Enchained growth and cluster dislocation: A possible mechanism for microbiota homeostasis. PLoS Comput. Biol..

[107] Kadaoui K.A., Corthesy B. (2007). Secretory IgA mediates bacterial translocation to dendritic cells in mouse Peyer’s patches with restriction to mucosal compartment. J. Immunol..

[108] Rey J., Garin N., Spertini F., Corthesy B. (2004). Targeting of secretory IgA to Peyer’s patch dendritic and T cells after transport by intestinal M cells. J. Immunol..

[109] Bunker J.J., Flynn T.M., Koval J.C., Shaw D.G., Meisel M., McDonald B.D., Ishizuka I.E., Dent A.L., Wilson P.C., Jabri B. (2015). Innate and Adaptive Humoral Responses Coat Distinct Commensal Bacteria with Immunoglobulin A. Immunity.

[110] Van der Waaij L.A., Limburg P.C., Mesander G., van der Waaij D. (1996). In vivo IgA coating of anaerobic bacteria in human faeces. Gut.

[111] Nakajima A., Vogelzang A., Maruya M., Miyajima M., Murata M., Son A., Kuwahara T., Tsuruyama T., Yamada S., Matsuura M. (2018). IgA regulates the composition and metabolic function of gut microbiota by promoting symbiosis between bacteria. J. Exp. Med..

[112] Donaldson G.P., Ladinsky M.S., Yu K.B., Sanders J.G., Yoo B.B., Chou W.C., Conner M.E., Earl A.M., Knight R., Bjorkman P.J. (2018). Gut microbiota utilize immunoglobulin A for mucosal colonization. Science.

[113] Briliute J., Urbanowicz P.A., Luis A.S., Basle A., Paterson N., Rebello O., Hendel J., Ndeh D.A., Lowe E.C., Martens E.C. (2019). Complex N-glycan breakdown by gut Bacteroides involves an extensive enzymatic apparatus encoded by multiple co-regulated genetic loci. Nat. Microbiol..

[114] Fehr K., Moossavi S., Sbihi H., Boutin R.C.T., Bode L., Robertson B., Yonemitsu C., Field C.J., Becker A.B., Mandhane P.J. (2020). Breastmilk Feeding Practices Are Associated with the Co-Occurrence of Bacteria in Mothers’ Milk and the Infant Gut: The CHILD Cohort Study. Cell Host Microbe..

[115] McLoughlin K., Schluter J., Rakoff-Nahoum S., Smith A.L., Foster K.R. (2016). Host Selection of Microbiota via Differential Adhesion. Cell Host Microbe.

[116] Kawamoto S., Maruya M., Kato L.M., Suda W., Atarashi K., Doi Y., Tsutsui Y., Qin H., Honda K., Okada T. (2014). Foxp3(+) T cells regulate immunoglobulin a selection and facilitate diversification of bacterial species responsible for immune homeostasis. Immunity.

[117] Atarashi K., Tanoue T., Oshima K., Suda W., Nagano Y., Nishikawa H., Fukuda S., Saito T., Narushima S., Hase K. (2013). Treg induction by a rationally selected mixture of Clostridia strains from the human microbiota. Nature.

[118] Atarashi K., Tanoue T., Shima T., Imaoka A., Kuwahara T., Momose Y., Cheng G., Yamasaki S., Saito T., Ohba Y. (2011). Induction of colonic regulatory T cells by indigenous Clostridium species. Science.

[119] Ansaldo E., Slayden L.C., Ching K.L., Koch M.A., Wolf N.K., Plichta D.R., Brown E.M., Graham D.B., Xavier R.J., Moon J.J. (2019). Akkermansia muciniphila induces intestinal adaptive immune responses during homeostasis. Science.

[120] Bunker J.J., Bendelac A. (2018). IgA Responses to Microbiota. Immunity.

[121] Blumberg R., Powrie F. (2012). Microbiota, disease, and back to health: A metastable journey. Sci. Transl. Med..

[122] Yel L. (2010). Selective IgA deficiency. J. Clin. Immunol..

[123] Jorgensen G.H., Thorsteinsdottir I., Gudmundsson S., Hammarstrom L., Ludviksson B.R. (2009). Familial aggregation of IgAD and autoimmunity. Clin. Immunol.

[124] Jacob C.M., Pastorino A.C., Fahl K., Carneiro-Sampaio M., Monteiro R.C. (2008). Autoimmunity in IgA deficiency: Revisiting the role of IgA as a silent housekeeper. J. Clin. Immunol..

[125] Shulzhenko N., Morgun A., Hsiao W., Battle M., Yao M., Gavrilova O., Orandle M., Mayer L., Macpherson A.J., McCoy K.D. (2011). Crosstalk between B lymphocytes, microbiota and the intestinal epithelium governs immunity versus metabolism in the gut. Nat. Med..

[126] Fadlallah J., El Kafsi H., Sterlin D., Juste C., Parizot C., Dorgham K., Autaa G., Gouas D., Almeida M., Lepage P. (2018). Microbial ecology perturbation in human IgA deficiency. Sci Transl Med..

[127] Sterlin D., Fieschi C., Malphettes M., Larsen M., Gorochov G., Fadlallah J. (2019). Immune/microbial interface perturbation in human IgA deficiency. Gut Microbes.

[128] Catanzaro J.R., Strauss J.D., Bielecka A., Porto A.F., Lobo F.M., Urban A., Schofield W.B., Palm N.W. (2019). IgA-deficient humans exhibit gut microbiota dysbiosis despite secretion of compensatory IgM. Sci. Rep..

[129] Fadlallah J., Sterlin D., Fieschi C., Parizot C., Dorgham K., El Kafsi H., Autaa G., Ghillani-Dalbin P., Juste C., Lepage P. (2019). Synergistic convergence of microbiota-specific systemic IgG and secretory IgA. J. Allergy Clin. Immunol..

[130] Moll J.M., Myers P.N., Zhang C., Eriksen C., Wolf J., Appelberg K.S., Lindberg G., Bahl M.I., Zhao H., Pan-Hammarstrom Q. (2021). Gut Microbiota Perturbation in IgA Deficiency Is Influenced by IgA-Autoantibody Status. Gastroenterology.

[131] Nagaishi T., Watabe T., Kotake K., Kumazawa T., Aida T., Tanaka K., Ono R., Ishino F., Usami T., Miura T. (2021). Immunoglobulin A-specific deficiency induces spontaneous inflammation specifically in the ileum. Gut.

[132] Hammarstrom L., Vorechovsky I., Webster D. (2000). Selective IgA deficiency (SIgAD) and common variable immunodeficiency (CVID). Clin. Exp. Immunol..

[133] Jorgensen S.F., Troseid M., Kummen M., Anmarkrud J.A., Michelsen A.E., Osnes L.T., Holm K., Hoivik M.L., Rashidi A., Dahl C.P. (2016). Altered gut microbiota profile in common variable immunodeficiency associates with levels of lipopolysaccharide and markers of systemic immune activation. Mucosal. Immunol..

[134] Rigoni R., Fontana E., Guglielmetti S., Fosso B., D’Erchia A.M., Maina V., Taverniti V., Castiello M.C., Mantero S., Pacchiana G. (2016). Intestinal microbiota sustains inflammation and autoimmunity induced by hypomorphic RAG defects. J. Exp. Med..

[135] Mohammed A.D., Khan M.A.W., Chatzistamou I., Chamseddine D., Williams-Kang K., Perry M., Enos R., Murphy A., Gomez G., Aladhami A. (2019). Gut Antibody Deficiency in a Mouse Model of CVID Results in Spontaneous Development of a Gluten-Sensitive Enteropathy. Front. Immunol..

[136] Shulzhenko N., Dong X., Vyshenska D., Greer R.L., Gurung M., Vasquez-Perez S., Peremyslova E., Sosnovtsev S., Quezado M., Yao M. (2018). CVID enteropathy is characterized by exceeding low mucosal IgA levels and interferon-driven inflammation possibly related to the presence of a pathobiont. Clin. Immunol..

[137] Neu J., Walker W.A. (2011). Necrotizing enterocolitis. N. Engl. J. Med..

[138] Gephart S.M., McGrath J.M., Effken J.A., Halpern M.D. (2012). Necrotizing enterocolitis risk: State of the science. Adv. Neonatal. Care.

[139] Milani C., Duranti S., Bottacini F., Casey E., Turroni F., Mahony J., Belzer C., Delgado Palacio S., Arboleya Montes S., Mancabelli L. (2017). The First Microbial Colonizers of the Human Gut: Composition, Activities, and Health Implications of the Infant Gut Microbiota. Microbiol. Mol. Biol. Rev..

[140] Zhou Y., Shan G., Sodergren E., Weinstock G., Walker W.A., Gregory K.E. (2015). Longitudinal analysis of the premature infant intestinal microbiome prior to necrotizing enterocolitis: A case-control study. PLoS ONE.

[141] Alexander V.N., Northrup V., Bizzarro M.J. (2011). Antibiotic exposure in the newborn intensive care unit and the risk of necrotizing enterocolitis. J. Pediatr..

[142] Berger J.N., Gong H., Good M., McElroy S.J. (2019). Dithizone-induced Paneth cell disruption significantly decreases intestinal perfusion in the murine small intestine. J. Pediatr. Surg..

[143] Lueschow S.R., Stumphy J., Gong H., Kern S.L., Elgin T.G., Underwood M.A., Kalanetra K.M., Mills D.A., Wong M.H., Meyerholz D.K. (2018). Loss of murine Paneth cell function alters the immature intestinal microbiome and mimics changes seen in neonatal necrotizing enterocolitis. PLoS ONE.

[144] White J.R., Gong H., Pope B., Schlievert P., McElroy S.J. (2017). Paneth-cell-disruption-induced necrotizing enterocolitis in mice requires live bacteria and occurs independently of TLR4 signaling. Dis. Model. Mech..

[145] Musemeche C.A., Kosloske A.M., Bartow S.A., Umland E.T. (1986). Comparative effects of ischemia, bacteria, and substrate on the pathogenesis of intestinal necrosis. J. Pediatr. Surg..

[146] Itani T., Ayoub Moubareck C., Melki I., Rousseau C., Mangin I., Butel M.J., Karam-Sarkis D. (2018). Preterm infants with necrotising enterocolitis demonstrate an unbalanced gut microbiota. Acta Paediatr..

[147] Paveglio S., Ledala N., Rezaul K., Lin Q., Zhou Y., Provatas A.A., Bennett E., Lindberg T., Caimano M., Matson A.P. (2020). Cytotoxin-producing Klebsiella oxytoca in the preterm gut and its association with necrotizing enterocolitis. Emerg. Microbes Infect..

[148] Olm M.R., Bhattacharya N., Crits-Christoph A., Firek B.A., Baker R., Song Y.S., Morowitz M.J., Banfield J.F. (2019). Necrotizing enterocolitis is preceded by increased gut bacterial replication, Klebsiella, and fimbriae-encoding bacteria. Sci. Adv..

[149] Shaw A.G., Sim K., Rose G., Wooldridge D.J., Li M.S., Misra R.V., Gharbia S., Kroll J.S. (2021). Premature neonatal gut microbial community patterns supporting an epithelial TLR-mediated pathway for necrotizing enterocolitis. BMC Microbiol..

[150] McMurtry V.E., Gupta R.W., Tran L., Blanchard E.E.T., Penn D., Taylor C.M., Ferris M.J. (2015). Bacterial diversity and Clostridia abundance decrease with increasing severity of necrotizing enterocolitis. Microbiome.

[151] La Rosa P.S., Warner B.B., Zhou Y., Weinstock G.M., Sodergren E., Hall-Moore C.M., Stevens H.J., Bennett W.E., Shaikh N., Linneman L.A. (2014). Patterned progression of bacterial populations in the premature infant gut. Proc. Natl. Acad. Sci. USA.

[152] Lueschow S.R., Kern S.L., Gong H., Grobe J.L., Segar J.L., Carlson S.J., McElroy S.J. (2020). Feeding Formula Eliminates the Necessity of Bacterial Dysbiosis and Induces Inflammation and Injury in the Paneth Cell Disruption Murine NEC Model in an Osmolality-Dependent Manner. Nutrients.

[153] Cortez J., Makker K., Kraemer D.F., Neu J., Sharma R., Hudak M.L. (2018). Maternal milk feedings reduce sepsis, necrotizing enterocolitis and improve outcomes of premature infants. J. Perinatol..

[154] Parr E.L., Bozzola J.J., Parr M.B. (1995). Purification and measurement of secretory IgA in mouse milk. J. Immunol. Methods.

[155] Gopalakrishna K.P., Macadangdang B.R., Rogers M.B., Tometich J.T., Firek B.A., Baker R., Ji J., Burr A.H.P., Ma C., Good M. (2019). Maternal IgA protects against the development of necrotizing enterocolitis in preterm infants. Nat. Med..

[156] Mirpuri J., Raetz M., Sturge C.R., Wilhelm C.L., Benson A., Savani R.C., Hooper L.V., Yarovinsky F. (2014). Proteobacteria-specific IgA regulates maturation of the intestinal microbiota. Gut Microbes.

[157] Eibl M.M., Wolf H.M., Furnkranz H., Rosenkranz A. (1988). Prevention of necrotizing enterocolitis in low-birth-weight infants by IgA-IgG feeding. N. Engl. J. Med..

[158] Meng X., Dunsmore G., Koleva P., Elloumi Y., Wu R.Y., Sutton R.T., Ambrosio L., Hotte N., Nguyen V., Madsen K.L. (2019). The Profile of Human Milk Metabolome, Cytokines, and Antibodies in Inflammatory Bowel Diseases Versus Healthy Mothers, and Potential Impact on the Newborn. J. Crohns Colitis.

[159] Brawner K.M., Yeramilli V.A., Kennedy B.A., Patel R.K., Martin C.A. (2020). Prenatal stress increases IgA coating of offspring microbiota and exacerbates necrotizing enterocolitis-like injury in a sex-dependent manner. Brain Behav. Immun..

[160] Zhang K., Zhang X., Lv A., Fan S., Zhang J. (2020). Saccharomyces boulardii modulates necrotizing enterocolitis in neonatal mice by regulating the sirtuin 1/NFkappaB pathway and the intestinal microbiota. Mol. Med. Rep..

[161] Morgan R.L., Preidis G.A., Kashyap P.C., Weizman A.V., Sadeghirad B., McMaster Probiotic P., Synbiotic Work G. (2020). Probiotics Reduce Mortality and Morbidity in Preterm, Low-Birth-Weight Infants: A Systematic Review and Network Meta-analysis of Randomized Trials. Gastroenterology.

[162] Isani M., Bell B.A., Delaplain P.T., Bowling J.D., Golden J.M., Elizee M., Illingworth L., Wang J., Gayer C.P., Grishin A.V. (2018). Lactobacillus murinus HF12 colonizes neonatal gut and protects rats from necrotizing enterocolitis. PLoS ONE.

[163] Colombel J.F., Mahadevan U. (2017). Inflammatory Bowel Disease 2017: Innovations and Changing Paradigms. Gastroenterology.

[164] Alatab S., Sepanlou S.G., Ikuta K., Vahedi H., Bisignano C., Safiri S., Sadeghi A., Nixon M.R., Abdoli A., Abolhassani H. (2020). The global, regional, and national burden of inflammatory bowel disease in 195 countries and territories, 1990–2017: A systematic analysis for the Global Burden of Disease Study 2017. Lancet Gastroenterol. Hepatol..

[165] Hernandez-Chirlaque C., Aranda C.J., Ocon B., Capitan-Canadas F., Ortega-Gonzalez M., Carrero J.J., Suarez M.D., Zarzuelo A., Sanchez de Medina F., Martinez-Augustin O. (2016). Germ-free and Antibiotic-treated Mice are Highly Susceptible to Epithelial Injury in DSS Colitis. J. Crohns Colitis.

[166] Jangid A., Fukuda S., Seki M., Horiuchi T., Suzuki Y., Taylor T.D., Ohno H., Prakash T. (2020). Association of colitis with gut-microbiota dysbiosis in clathrin adapter AP-1B knockout mice. PLoS ONE.

[167] Selvanantham T., Lin Q., Guo C.X., Surendra A., Fieve S., Escalante N.K., Guttman D.S., Streutker C.J., Robertson S.J., Philpott D.J. (2016). NKT Cell-Deficient Mice Harbor an Altered Microbiota That Fuels Intestinal Inflammation during Chemically Induced Colitis. J. Immunol..

[168] Zhang Q., Wu Y., Wang J., Wu G., Long W., Xue Z., Wang L., Zhang X., Pang X., Zhao Y. (2016). Accelerated dysbiosis of gut microbiota during aggravation of DSS-induced colitis by a butyrate-producing bacterium. Sci. Rep..

[169] Garrett W.S., Gallini C.A., Yatsunenko T., Michaud M., DuBois A., Delaney M.L., Punit S., Karlsson M., Bry L., Glickman J.N. (2010). Enterobacteriaceae act in concert with the gut microbiota to induce spontaneous and maternally transmitted colitis. Cell Host Microbe.

[170] Lupp C., Robertson M.L., Wickham M.E., Sekirov I., Champion O.L., Gaynor E.C., Finlay B.B. (2007). Host-mediated inflammation disrupts the intestinal microbiota and promotes the overgrowth of Enterobacteriaceae. Cell Host Microbe.

[171] Imhann F., Vich Vila A., Bonder M.J., Fu J., Gevers D., Visschedijk M.C., Spekhorst L.M., Alberts R., Franke L., van Dullemen H.M. (2018). Interplay of host genetics and gut microbiota underlying the onset and clinical presentation of inflammatory bowel disease. Gut.

[172] Reikvam D.H., Derrien M., Islam R., Erofeev A., Grcic V., Sandvik A., Gaustad P., Meza-Zepeda L.A., Jahnsen F.L., Smidt H. (2012). Epithelial-microbial crosstalk in polymeric Ig receptor deficient mice. Eur. J. Immunol..

[173] Landuyt A.E., Klocke B.J., Duck L.W., Kemp K.M., Muir R.Q., Jennings M.S., Blum S.I., Tse H.M., Lee G., Morrow C.D. (2021). ICOS ligand and IL-10 synergize to promote host-microbiota mutualism. Proc. Natl. Acad. Sci. USA.

[174] Cao Y., Wang X., Yang Q., Deng H., Liu Y., Zhou P., Xu H., Chen D., Feng D., Zhang H. (2020). Critical Role of Intestinal Microbiota in ATF3-Mediated Gut Immune Homeostasis. J. Immunol..

[175] Cao Y., Yang Q., Deng H., Tang J., Hu J., Liu H., Zhi M., Ye L., Zou B., Liu Y. (2019). Transcriptional factor ATF3 protects against colitis by regulating follicular helper T cells in Peyer’s patches. Proc. Natl. Acad. Sci. USA.

[176] Kubinak J.L., Petersen C., Stephens W.Z., Soto R., Bake E., O’Connell R.M., Round J.L. (2015). MyD88 signaling in T cells directs IgA-mediated control of the microbiota to promote health. Cell Host Microbe.

[177] Wang S., Charbonnier L.M., Noval Rivas M., Georgiev P., Li N., Gerber G., Bry L., Chatila T.A. (2015). MyD88 Adaptor-Dependent Microbial Sensing by Regulatory T Cells Promotes Mucosal Tolerance and Enforces Commensalism. Immunity.

[178] Pascual-Itoiz M.A., Pena-Cearra A., Martin-Ruiz I., Lavin J.L., Simo C., Rodriguez H., Atondo E., Flores J.M., Carreras-Gonzalez A., Tomas-Cortazar J. (2020). The mitochondrial negative regulator MCJ modulates the interplay between microbiota and the host during ulcerative colitis. Sci. Rep..

[179] Harrington L., Srikanth C.V., Antony R., Rhee S.J., Mellor A.L., Shi H.N., Cherayil B.J. (2008). Deficiency of indoleamine 2,3-dioxygenase enhances commensal-induced antibody responses and protects against Citrobacter rodentium-induced colitis. Infect. Immun..

[180] Van der Waaij L.A., Kroese F.G., Visser A., Nelis G.F., Westerveld B.D., Jansen P.L., Hunter J.O. (2004). Immunoglobulin coating of faecal bacteria in inflammatory bowel disease. Eur. J. Gastroenterol. Hepatol..

[181] Palm N.W., de Zoete M.R., Cullen T.W., Barry N.A., Stefanowski J., Hao L., Degnan P.H., Hu J., Peter I., Zhang W. (2014). Immunoglobulin A coating identifies colitogenic bacteria in inflammatory bowel disease. Cell.

[182] Jackson M.A., Pearson C., Ilott N.E., Huus K.E., Hegazy A.N., Webber J., Finlay B.B., Macpherson A.J., Powrie F., Lam L.H. (2021). Accurate identification and quantification of commensal microbiota bound by host immunoglobulins. Microbiome.

[183] Shapiro J.M., de Zoete M.R., Palm N.W., Laenen Y., Bright R., Mallette M., Bu K., Bielecka A.A., Xu F., Hurtado-Lorenzo A. (2021). Immunoglobulin A Targets a Unique Subset of the Microbiota in Inflammatory Bowel Disease. Cell Host Microbe.

[184] Lin R., Chen H., Shu W., Sun M., Fang L., Shi Y., Pang Z., Wu W., Liu Z. (2018). Clinical significance of soluble immunoglobulins A and G and their coated bacteria in feces of patients with inflammatory bowel disease. J. Transl. Med..

[185] Gupta S., Basu S., Bal V., Rath S., George A. (2019). Gut IgA abundance in adult life is a major determinant of resistance to dextran sodium sulfate-colitis and can compensate for the effects of inadequate maternal IgA received by neonates. Immunology.

[186] Rochereau N., Roblin X., Michaud E., Gayet R., Chanut B., Jospin F., Corthesy B., Paul S. (2021). NOD2 deficiency increases retrograde transport of secretory IgA complexes in Crohn’s disease. Nat. Commun..

[187] Zhang T., Ding C., Zhao M., Dai X., Yang J., Li Y., Gu L., Wei Y., Gong J., Zhu W. (2016). Sodium Butyrate Reduces Colitogenic Immunoglobulin A-Coated Bacteria and Modifies the Composition of Microbiota in IL-10 Deficient Mice. Nutrients.

[188] Okai S., Usui F., Ohta M., Mori H., Kurokawa K., Matsumoto S., Kato T., Miyauchi E., Ohno H., Shinkura R. (2017). Intestinal IgA as a modulator of the gut microbiota. Gut Microbes.

[189] Rogier E.W., Frantz A.L., Bruno M.E., Wedlund L., Cohen D.A., Stromberg A.J., Kaetzel C.S. (2014). Secretory antibodies in breast milk promote long-term intestinal homeostasis by regulating the gut microbiota and host gene expression. Proc. Natl. Acad. Sci. USA.

[190] Ramanan D., Sefik E., Galvan-Pena S., Wu M., Yang L., Yang Z., Kostic A., Golovkina T.V., Kasper D.L., Mathis D. (2020). An Immunologic Mode of Multigenerational Transmission Governs a Gut Treg Setpoint. Cell.

[191] Siegel R.L., Miller K.D., Fuchs H.E., Jemal A. (2021). Cancer Statistics, 2021. CA Cancer J. Clin..

[192] Chalkias A., Nikotian G., Koutsovasilis A., Bramis J., Manouras A., Mystrioti D., Katergiannakis V. (2011). Patients with colorectal cancer are characterized by increased concentration of fecal hb-hp complex, myeloperoxidase, and secretory IgA. Am. J. Clin. Oncol..

[193] Chen M., Lin X., Zhang L., Yu L., Wu Q., Zhang S., Xue F., Huang Y. (2020). Development of a panel of serum IgG and IgA autoantibodies for early diagnosis of colon cancer. Int. J. Med. Sci.

[194] De Chiara L., Paez de la Cadena M., Rodriguez-Berrocal J., Alvarez-Pardinas M.C., Pardinas-Anon M.C., Varela-Calvino R., Cordero O.J. (2020). CD26-Related Serum Biomarkers: sCD26 Protein, DPP4 Activity, and Anti-CD26 Isotype Levels in a Colorectal Cancer-Screening Context. Dis. Markers.

[195] Butvilovskaya V.I., Popletaeva S.B., Chechetkin V.R., Zubtsova Z.I., Tsybulskaya M.V., Samokhina L.O., Vinnitskii L.I., Ragimov A.A., Pozharitskaya E.I., Grigor Eva G.A. (2016). Multiplex determination of serological signatures in the sera of colorectal cancer patients using hydrogel biochips. Cancer Med..

[196] Staff C., Magnusson C.G., Hojjat-Farsangi M., Mosolits S., Liljefors M., Frodin J.E., Wahren B., Mellstedt H., Ullenhag G.J. (2012). Induction of IgM, IgA and IgE antibodies in colorectal cancer patients vaccinated with a recombinant CEA protein. J. Clin. Immunol..

[197] Kurt M., Yumuk Z. (2021). Diagnostic accuracy of Fusobacterium nucleatum IgA and IgG ELISA test in colorectal cancer. Sci. Rep..

[198] Magat E.M., Balanag G.A., CariNo A.M., Fellizar A., Ortin T.S., Guevarra L., Albano P.M. (2020). Clostridioides difficile antibody response of colorectal cancer patients versus clinically healthy individuals. Biosci. Microbiota Food Health.

[199] Wang H.F., Li L.F., Guo S.H., Zeng Q.Y., Ning F., Liu W.L., Zhang G. (2016). Evaluation of antibody level against Fusobacterium nucleatum in the serological diagnosis of colorectal cancer. Sci. Rep..

[200] Mion F., Vetrano S., Tonon S., Valeri V., Piontini A., Burocchi A., Petti L., Frossi B., Gulino A., Tripodo C. (2017). Reciprocal influence of B cells and tumor macro and microenvironments in the Apc(Min/+) model of colorectal cancer. Oncoimmunology.

[201] Wang W., Zhong Y., Zhuang Z., Xie J., Lu Y., Huang C., Sun Y., Wu L., Yin J., Yu H. (2021). Multiregion single-cell sequencing reveals the transcriptional landscape of the immune microenvironment of colorectal cancer. Clin. Transl. Med..

[202] Hale L.P. (2020). Deficiency of activation-induced cytidine deaminase in a murine model of ulcerative colitis. PLoS ONE.

[203] Muthuswamy R.V., Sundstrom P., Borjesson L., Gustavsson B., Quiding-Jarbrink M. (2013). Impaired migration of IgA-secreting cells to colon adenocarcinomas. Cancer Immunol. Immunother.

[204] Malik A., Sharma D., Zhu Q., Karki R., Guy C.S., Vogel P., Kanneganti T.D. (2016). IL-33 regulates the IgA-microbiota axis to restrain IL-1alpha-dependent colitis and tumorigenesis. J. Clin. Investig..

[205] Garrett W.S. (2019). The gut microbiota and colon cancer. Science.

[206] Berger J., Hinglais N. (1968). [Intercapillary deposits of IgA-IgG]. J. Urol. Nephrol..

[207] Tomana M., Novak J., Julian B.A., Matousovic K., Konecny K., Mestecky J. (1999). Circulating immune complexes in IgA nephropathy consist of IgA1 with galactose-deficient hinge region and antiglycan antibodies. J. Clin. Investig..

[208] Berger J., Yaneva H., Nabarra B., Barbanel C. (1975). Recurrence of mesangial deposition of IgA after renal transplantation. Kidney Int.

[209] Berger J. (1969). IgA glomerular deposits in renal disease. Transplant. Proc..

[210] Zhang Y.M., Zhang H. (2018). Insights into the Role of Mucosal Immunity in IgA Nephropathy. Clin. J. Am. Soc. Nephrol.

[211] Rodrigues J.C., Haas M., Reich H.N. (2017). IgA Nephropathy. Clin. J. Am. Soc. Nephrol..

[212] Jarrick S., Lundberg S., Welander A., Carrero J.J., Hoijer J., Bottai M., Ludvigsson J.F. (2019). Mortality in IgA Nephropathy: A Nationwide Population-Based Cohort Study. J. Am. Soc. Nephrol..

[213] Sallustio F., Curci C., Chaoul N., Fonto G., Lauriero G., Picerno A., Divella C., Di Leo V., De Angelis M., Ben Mkaddem S. (2021). High levels of gut-homing immunoglobulin A+ B lymphocytes support the pathogenic role of intestinal mucosal hyperresponsiveness in immunoglobulin A nephropathy patients. Nephrol. Dial. Transplant..

[214] Kamata T., Nogaki F., Fagarasan S., Sakiyama T., Kobayashi I., Miyawaki S., Ikuta K., Muso E., Yoshida H., Sasayama S. (2000). Increased frequency of surface IgA-positive plasma cells in the intestinal lamina propria and decreased IgA excretion in hyper IgA (HIGA) mice, a murine model of IgA nephropathy with hyperserum IgA. J. Immunol..

[215] Kiryluk K., Moldoveanu Z., Sanders J.T., Eison T.M., Suzuki H., Julian B.A., Novak J., Gharavi A.G., Wyatt R.J. (2011). Aberrant glycosylation of IgA1 is inherited in both pediatric IgA nephropathy and Henoch-Schonlein purpura nephritis. Kidney Int..

[216] Gharavi A.G., Moldoveanu Z., Wyatt R.J., Barker C.V., Woodford S.Y., Lifton R.P., Mestecky J., Novak J., Julian B.A. (2008). Aberrant IgA1 glycosylation is inherited in familial and sporadic IgA nephropathy. J. Am. Soc. Nephrol..

[217] Zhai Y.L., Zhu L., Shi S.F., Liu L.J., Lv J.C., Zhang H. (2016). Increased APRIL Expression Induces IgA1 Aberrant Glycosylation in IgA Nephropathy. Medicine.

[218] Yu X.Q., Li M., Zhang H., Low H.Q., Wei X., Wang J.Q., Sun L.D., Sim K.S., Li Y., Foo J.N. (2011). A genome-wide association study in Han Chinese identifies multiple susceptibility loci for IgA nephropathy. Nat. Genet..

[219] McCarthy D.D., Kujawa J., Wilson C., Papandile A., Poreci U., Porfilio E.A., Ward L., Lawson M.A., Macpherson A.J., McCoy K.D. (2011). Mice overexpressing BAFF develop a commensal flora-dependent, IgA-associated nephropathy. J. Clin. Investig..

[220] McCarthy D.D., Chiu S., Gao Y., Summers-deLuca L.E., Gommerman J.L. (2006). BAFF induces a hyper-IgA syndrome in the intestinal lamina propria concomitant with IgA deposition in the kidney independent of LIGHT. Cell Immunol..

[221] Roos A., Rastaldi M.P., Calvaresi N., Oortwijn B.D., Schlagwein N., van Gijlswijk-Janssen D.J., Stahl G.L., Matsushita M., Fujita T., van Kooten C. (2006). Glomerular activation of the lectin pathway of complement in IgA nephropathy is associated with more severe renal disease. J. Am. Soc. Nephrol..

[222] Roos A., Bouwman L.H., van Gijlswijk-Janssen D.J., Faber-Krol M.C., Stahl G.L., Daha M.R. (2001). Human IgA activates the complement system via the mannan-binding lectin pathway. J. Immunol..

[223] Huang Z.Q., Raska M., Stewart T.J., Reily C., King R.G., Crossman D.K., Crowley M.R., Hargett A., Zhang Z., Suzuki H. (2016). Somatic Mutations Modulate Autoantibodies against Galactose-Deficient IgA1 in IgA Nephropathy. J. Am. Soc. Nephrol..

[224] Kiryluk K., Li Y., Scolari F., Sanna-Cherchi S., Choi M., Verbitsky M., Fasel D., Lata S., Prakash S., Shapiro S. (2014). Discovery of new risk loci for IgA nephropathy implicates genes involved in immunity against intestinal pathogens. Nat. Genet..

[225] He J.W., Zhou X.J., Li Y.F., Wang Y.N., Liu L.J., Shi S.F., Xin X.H., Li R.S., Falchi M., Lv J.C. (2021). Associations of Genetic Variants Contributing to Gut Microbiota Composition in Immunoglobin A Nephropathy. mSystems.

[226] Dong R., Bai M., Zhao J., Wang D., Ning X., Sun S. (2020). A Comparative Study of the Gut Microbiota Associated With Immunoglobulin a Nephropathy and Membranous Nephropathy. Front. Cell Infect. Microbiol.

[227] Chai L., Luo Q., Cai K., Wang K., Xu B. (2021). Reduced fecal short-chain fatty acids levels and the relationship with gut microbiota in IgA nephropathy. BMC Nephrol..

[228] Chemouny J.M., Gleeson P.J., Abbad L., Lauriero G., Boedec E., Le Roux K., Monot C., Bredel M., Bex-Coudrat J., Sannier A. (2019). Modulation of the microbiota by oral antibiotics treats immunoglobulin A nephropathy in humanized mice. Nephrol. Dial. Transplant..

[229] Lamm M.E., Emancipator S.N., Robinson J.K., Yamashita M., Fujioka H., Qiu J., Plaut A.G. (2008). Microbial IgA protease removes IgA immune complexes from mouse glomeruli in vivo: Potential therapy for IgA nephropathy. Am. J. Pathol.

[230] Pillebout E. (2021). IgA Vasculitis and IgA Nephropathy: Same Disease?. J. Clin. Med..

[231] Wang X., Zhang L., Wang Y., Liu X., Zhang H., Liu Y., Shen N., Yang J., Gai Z. (2018). Gut microbiota dysbiosis is associated with Henoch-Schonlein Purpura in children. Int. Immunopharmacol..

[232] Zhang Y., Xia G., Nie X., Zeng Y., Chen Y., Qian Y., Chen G., Huang J., Wang C., Zhang C. (2021). Differences in Manifestations and Gut Microbiota Composition Between Patients With Different Henoch-Schonlein Purpura Phenotypes. Front. Cell Infect. Microbiol..

[233] Khan I., Li X.A., Law B., U K.I., Pan B.Q., Lei C., Hsiao W.W. (2020). Correlation of gut microbial compositions to the development of Kawasaki disease vasculitis in children. Future Microbiol..

[234] Ye Z., Zhang N., Wu C., Zhang X., Wang Q., Huang X., Du L., Cao Q., Tang J., Zhou C. (2018). A metagenomic study of the gut microbiome in Behcet’s disease. Microbiome.

[235] Shimizu J., Kubota T., Takada E., Takai K., Fujiwara N., Arimitsu N., Ueda Y., Wakisaka S., Suzuki T., Suzuki N. (2016). Bifidobacteria Abundance-Featured Gut Microbiota Compositional Change in Patients with Behcet’s Disease. PLoS ONE.

[236] Consolandi C., Turroni S., Emmi G., Severgnini M., Fiori J., Peano C., Biagi E., Grassi A., Rampelli S., Silvestri E. (2015). Behcet’s syndrome patients exhibit specific microbiome signature. Autoimmun Rev..

[237] Ajmera A., Shabbir N. (2021). Salmonella.

[238] Boore A.L., Hoekstra R.M., Iwamoto M., Fields P.I., Bishop R.D., Swerdlow D.L. (2015). Salmonella enterica Infections in the United States and Assessment of Coefficients of Variation: A Novel Approach to Identify Epidemiologic Characteristics of Individual Serotypes, 1996–2011. PLoS ONE.

[239] Wotzka S.Y., Nguyen B.D., Hardt W.D. (2017). Salmonella Typhimurium Diarrhea Reveals Basic Principles of Enteropathogen Infection and Disease-Promoted DNA Exchange. Cell Host Microbe.

[240] Jones B.D., Ghori N., Falkow S. (1994). Salmonella typhimurium initiates murine infection by penetrating and destroying the specialized epithelial M cells of the Peyer’s patches. J. Exp. Med..

[241] Hashizume-Takizawa T., Kobayashi R., Tsuzukibashi O., Saito M., Kurita-Ochiai T. (2019). CCR7-deficient mice exhibit a delayed antigen-specific mucosal IgA antibody response to an oral recombinant Salmonella strain. Pathog. Dis..

[242] Martinoli C., Chiavelli A., Rescigno M. (2007). Entry route of Salmonella typhimurium directs the type of induced immune response. Immunity.

[243] Vazquez-Torres A., Jones-Carson J., Baumler A.J., Falkow S., Valdivia R., Brown W., Le M., Berggren R., Parks W.T., Fang F.C. (1999). Extraintestinal dissemination of Salmonella by CD18-expressing phagocytes. Nature.

[244] Man A.L., Gicheva N., Regoli M., Rowley G., De Cunto G., Wellner N., Bassity E., Gulisano M., Bertelli E., Nicoletti C. (2017). CX3CR1+ Cell-Mediated Salmonella Exclusion Protects the Intestinal Mucosa during the Initial Stage of Infection. J. Immunol..

[245] Chami B., Yeung A., Buckland M., Liu H., Fong G.M., Tao K., Bao S. (2017). CXCR3 plays a critical role for host protection against Salmonellosis. Sci. Rep..

[246] Hashizume-Takizawa T., Shibata N., Kurashima Y., Kiyono H., Kurita-Ochiai T., Fujihashi K. (2019). Distinct roles for Peyer’s patch B cells for induction of antigen-specific IgA antibody responses in mice administered oral recombinant Salmonella. Int. Immunol..

[247] Hashizume T., Togawa A., Nochi T., Igarashi O., Kweon M.N., Kiyono H., Yamamoto M. (2008). Peyer’s patches are required for intestinal immunoglobulin A responses to Salmonella spp.. Infect. Immun..

[248] Richards A.F., Doering J.E., Lozito S.A., Varrone J.J., Willsey G.G., Pauly M., Whaley K., Zeitlin L., Mantis N.J. (2020). Inhibition of invasive salmonella by orally administered IgA and IgG monoclonal antibodies. PLoS Negl. Trop. Dis..

[249] Richards A.F., Baranova D.E., Pizzuto M.S., Jaconi S., Willsey G.G., Torres-Velez F.J., Doering J.E., Benigni F., Corti D., Mantis N.J. (2021). Recombinant Human Secretory IgA Induces Salmonella Typhimurium Agglutination and Limits Bacterial Invasion into Gut-Associated Lymphoid Tissues. ACS Infect. Dis..

[250] Bioley G., Monnerat J., Lotscher M., Vonarburg C., Zuercher A., Corthesy B. (2017). Plasma-Derived Polyreactive Secretory-Like IgA and IgM Opsonizing Salmonella enterica Typhimurium Reduces Invasion and Gut Tissue Inflammation through Agglutination. Front. Immunol..

[251] Corthesy B., Monnerat J., Lotscher M., Vonarburg C., Schaub A., Bioley G. (2018). Oral Passive Immunization with Plasma-Derived Polyreactive Secretory-Like IgA/M Partially Protects Mice Against Experimental Salmonellosis. Front. Immunol..

[252] Betz K.J., Maier E.A., Amarachintha S., Wu D., Karmele E.P., Kinder J.M., Steinbrecher K.A., McNeal M.M., Luzader D.H., Hogan S.P. (2018). Enhanced survival following oral and systemic Salmonella enterica serovar Typhimurium infection in polymeric immunoglobulin receptor knockout mice. PLoS ONE.

[253] Wijburg O.L., Uren T.K., Simpfendorfer K., Johansen F.E., Brandtzaeg P., Strugnell R.A. (2006). Innate secretory antibodies protect against natural Salmonella typhimurium infection. J. Exp. Med..

[254] Uren T.K., Wijburg O.L., Simmons C., Johansen F.E., Brandtzaeg P., Strugnell R.A. (2005). Vaccine-induced protection against gastrointestinal bacterial infections in the absence of secretory antibodies. Eur. J. Immunol..

[255] Zhao X., Zeng X., Dai Q., Hou Y., Zhu D., Wang M., Jia R., Chen S., Liu M., Yang Q. (2021). Immunogenicity and protection efficacy of a Salmonella enterica serovar Typhimurium fnr, arcA and fliC mutant. Vaccine.

[256] Endt K., Stecher B., Chaffron S., Slack E., Tchitchek N., Benecke A., Van Maele L., Sirard J.C., Mueller A.J., Heikenwalder M. (2010). The microbiota mediates pathogen clearance from the gut lumen after non-typhoidal Salmonella diarrhea. PLoS Pathog..

[257] LaRusso N.F. (1984). Proteins in bile: How they get there and what they do. Am. J. Physiol..

[258] Vuitton D.A., Seilles E., Claude P., Sava P., Delacroix D.L. (1985). Gall bladder: The predominant source of bile IgA in man?. Clin. Exp. Immunol..

[259] Wu C.T., Davis P.A., Luketic V.A., Gershwin M.E. (2004). A review of the physiological and immunological functions of biliary epithelial cells: Targets for primary biliary cirrhosis, primary sclerosing cholangitis and drug-induced ductopenias. Clin. Dev. Immunol..

[260] Brown W.R., Kloppel T.M. (1989). The role of the liver in translocation of IgA into the gastrointestinal tract. Immunol. Investig..

[261] Chandy K.G., Hubscher S.G., Elias E., Berg J., Khan M., Burnett D. (1983). Dual role of the liver in regulating circulating polymeric IgA in man: Studies on patients with liver disease. Clin. Exp. Immunol..

[262] Sakisaka S., Gondo K., Yoshitake M., Harada M., Sata M., Kobayashi K., Tanikawa K. (1996). Functional differences between hepatocytes and biliary epithelial cells in handling polymeric immunoglobulin A2 in humans, rats, and guinea pigs. Hepatology.

[263] Russell M.W., Brown T.A., Claflin J.L., Schroer K., Mestecky J. (1983). Immunoglobulin A-mediated hepatobiliary transport constitutes a natural pathway for disposing of bacterial antigens. Infect. Immun..

[264] Russell M.W., Brown T.A., Mestecky J. (1981). Role of serum IgA. Hepatobiliary transport of circulating antigen. J. Exp. Med..

[265] Counihan N.A., Anderson D.A. (2016). Specific IgA Enhances the Transcytosis and Excretion of Hepatitis A Virus. Sci. Rep..

[266] Haswell-Elkins M.R., Sithithaworn P., Mairiang E., Elkins D.B., Wongratanacheewin S., Kaewkes S., Mairiang P. (1991). Immune responsiveness and parasite-specific antibody levels in human hepatobiliary disease associated with Opisthorchis viverrini infection. Clin. Exp. Immunol..

[267] Jacob C.O., Vaerman J.P. (1986). Induction of rat secretory IgA antibodies against cholera toxin by a synthetic peptide. Immunology.

[268] Sharma A.W., Mayrhofer G. (1988). Biliary antibody response in rats infected with rodent Giardia duodenalis isolates. Parasite Immunol..

[269] Verdon R., Polianski J., Grodet A., Garry L., Carbon C. (1998). Cryptosporidium parvum biliary tract infection in adult immunocompetent and immunosuppressed mice. J. Med. Microbiol..

[270] Wongratanacheewin S., Bunnag D., Vaeusorn N., Sirisinha S. (1988). Characterization of humoral immune response in the serum and bile of patients with opisthorchiasis and its application in immunodiagnosis. Am. J. Trop Med. Hyg..

[271] Aagaard B.D., Heyworth M.F., Oesterle A.L., Jones A.L., Way L.W. (1996). Intestinal immunisation with Escherichia coli protects rats against Escherichia coli induced cholangitis. Gut.

[272] Yio X.Y., Jin B.W., Yin F.Z., Li X.J. (1992). Bile secretory immunoglobulin A in biliary infection and cholelithiasis. Gastroenterology.

[273] James S.P., Jones E.A., Schafer D.F., Hoofnagle J.H., Varma R.R., Strober W. (1986). Selective immunoglobulin A deficiency associated with primary biliary cirrhosis in a family with liver disease. Gastroenterology.

[274] Danon Y.L., Dinari G., Garty B.Z., Horodniceanu C., Nitzan M., Grunebaum M. (1983). Cholelithiasis in children with immunoglobulin A deficiency: A new gastroenterologic syndrome. J. Pediatr. Gastroenterol. Nutr..

[275] Iwata K., Komatsu T., Watanabe M., Nishiya H., Kunii O. (1983). Significance of test for antibody-coated bacteria in biliary tract infection. Jpn. J. Exp. Med..

[276] Moro-Sibilot L., Blanc P., Taillardet M., Bardel E., Couillault C., Boschetti G., Traverse-Glehen A., Defrance T., Kaiserlian D., Dubois B. (2016). Mouse and Human Liver Contain Immunoglobulin A-Secreting Cells Originating From Peyer’s Patches and Directed Against Intestinal Antigens. Gastroenterology.

[277] Wu T., Zhang Z., Liu B., Hou D., Liang Y., Zhang J., Shi P. (2013). Gut microbiota dysbiosis and bacterial community assembly associated with cholesterol gallstones in large-scale study. BMC Genomics.

[278] Saltykova I.V., Petrov V.A., Logacheva M.D., Ivanova P.G., Merzlikin N.V., Sazonov A.E., Ogorodova L.M., Brindley P.J. (2016). Biliary Microbiota, Gallstone Disease and Infection with Opisthorchis felineus. PLoS Negl. Trop. Dis..

[279] Petrov V.A., Fernandez-Peralbo M.A., Derks R., Knyazeva E.M., Merzlikin N.V., Sazonov A.E., Mayboroda O.A., Saltykova I.V. (2020). Biliary Microbiota and Bile Acid Composition in Cholelithiasis. Biomed. Res. Int..

[280] Plieskatt J.L., Deenonpoe R., Mulvenna J.P., Krause L., Sripa B., Bethony J.M., Brindley P.J. (2013). Infection with the carcinogenic liver fluke Opisthorchis viverrini modifies intestinal and biliary microbiome. FASEB J..

[281] Baumann U., Miescher S., Vonarburg C. (2014). Immunoglobulin replacement therapy in antibody deficiency syndromes: Are we really doing enough?. Clin. Exp. Immunol..

[282] Newman M.J., Benani D.J. (2016). A review of blinatumomab, a novel immunotherapy. J. Oncol. Pharm. Pract..

[283] Romero D. (2021). Amivantamab is effective in NSCLC harbouring EGFR exon 20 insertions. Nat. Rev. Clin. Oncol.

[284] Sedykh S.E., Prinz V.V., Buneva V.N., Nevinsky G.A. (2018). Bispecific antibodies: Design, therapy, perspectives. Drug Des. Devel. Ther..

[285] Deyev S.M., Lebedenko E.N. (2009). Modern Technologies for Creating Synthetic Antibodies for Clinical application. Acta Naturae.

[286] Yin Q., Pi X., Jiang Y., Ren G., Liu Z., Liu H., Wang M., Sun W., Li S., Gao Z. (2021). An immuno-blocking agent targeting IL-1beta and IL-17A reduces the lesion of DSS-induced ulcerative colitis in mice. Inflammation.

[287] Peyrin-Biroulet L., Demarest S., Nirula A. (2019). Bispecific antibodies: The next generation of targeted inflammatory bowel disease therapies. Autoimmun. Rev..

[288] Vandenbroucke K., de Haard H., Beirnaert E., Dreier T., Lauwereys M., Huyck L., Van Huysse J., Demetter P., Steidler L., Remaut E. (2010). Orally administered L. lactis secreting an anti-TNF Nanobody demonstrate efficacy in chronic colitis. Mucosal. Immunol..

